# Abundance and distribution patterns of cetaceans and their overlap with vessel traffic in the Humboldt Current Ecosystem, Chile

**DOI:** 10.1038/s41598-022-14465-7

**Published:** 2022-06-23

**Authors:** Luis Bedriñana-Romano, Patricia M. Zarate, Rodrigo Hucke-Gaete, Francisco A. Viddi, Susannah J. Buchan, Ilia Cari, Ljubitza Clavijo, Robert Bello, Alexandre N. Zerbini

**Affiliations:** 1 Instituto de Ciencias Marinas y Limnológicas, Facultad de Ciencias, Universidad Austral de Chile, Valdivia, Chile; 2 NGO Centro Ballena Azul, 5090000 Valdivia, Chile; 3 Centro de Investigación Oceanográfica COPAS Coastal, Universidad de Concepción, 4070043 Concepción, Región del Bio Bio Chile; 4 Instituto de Fomento Pesquero, Departamento de Oceanografía y Medio Ambiente, Almirante Manuel Blanco Encalada 839, Valparaíso, Chile; 5 Centro de Investigación Oceanográfica COPAS Sur-Austral, Universidad de Concepción, 4070043 Concepción, Región del Bio Chile; 6 Centro de Estudios Avanzados en Zonas Áridas, Raúl Bitran 1305, 1700000 La Serena, Región del Coquimbo Chile; 7 Cooperative Institute for Climate, Ocean and Ecosystem Studies, University of Washington & Marine Mammal Laboratory Alaska Fisheries Science Center/NOAA, 7600 Sand Point Way NE, Seattle, WA USA; 8 Marine Ecology and Telemetry Research, 2468 Camp McKenzie Tr NW, Seabeck, WA 98380 USA; 9 Instituto Aqualie, Av. Dr. Paulo Japiassú Coelho, 714, Sala 206, Juiz de Fora, MG 36033-310 Brazil; 10 MigraMar, 2099 Westshore Rd, CA 94923 Bodega Bay, USA

**Keywords:** Ecology, Biogeography, Conservation biology, Ecological modelling

## Abstract

The Humboldt Current Ecosystem (HCE) is one of the most productive marine ecosystems, sustaining one of the largest fishing industries in the world. Although several species of cetaceans are known to inhabit these productive waters, quantitative assessments of their abundance and distribution patterns are scarce and patchy. Here, we present the first abundance and distribution estimates for fin whale (*Balaenoptera physalus*), southeast Pacific blue whales (*Balaenoptera musculus*), sperm whale (*Physeter macrocephalus*), dusky dolphin (*Lagenorhynchus obscurus*), and common dolphin (*Delphinus* spp.) in the entire Chilean portion of the HCE. Line transect surveys were conducted during 2016–2021 between 18° S and 41° S and up to ~ 200 km offshore, and data were analyzed using distance sampling methods. Group counts were modelled as a function of environmental variables using single step Bayesian Binomial N-mixture model (BNMM), which allows full uncertainty propagation between model components. By using spatially explicit predictions of cetacean densities and observed vessel densities in the HCE, we provide quantitative assessments on the relative probability of cetaceans encountering vessels (RPCEV). Dusky dolphin and fin whale showed the largest distribution overlap with industrial and artisanal fishery fleets. Our results highlight areas where effort should be prioritized to address the extant but unquantified negative interactions between vessels and cetaceans in Chilean HCE.

## Introduction

The Humboldt Current Ecosystem (HCE) extends from the Galapagos Archipelago in the north to mid latitudes around ~ 42° S^1^ in the southeast Pacific Ocean. This productive current sustains about 10% of global fish landing^2^ and is characterized by drastic regime shifts, driven by El Niño Southern Oscillation (ENSO), which modifies the entire ecosystem structure^1,3,4^. Despite the enormous scientific efforts dedicated to understanding the dynamics of planktonic communities^5–7^, intertidal benthic communities^3,8,9^, and economically relevant fish and invertebrate species^10–12^ within the HCE, the ecology of large marine megafauna, such as birds, cartilaginous fishes, mammals and turtles is poorly known^13–15^.

Among marine mammals, cetaceans in the HCE have been subject to small-scale (< 100 km) studies^16–18^. Quantitative assessments about their biogeographical patterns are scarce^19–21^ and only one study focusing in blue whales has provided an abundance estimate^22^. Abundance estimates are paramount for assessing population trends and dynamics^23,24^. In addition, when abundance is estimated using model-based approaches, spatial predictions on density or probability of occurrence can be used for spatially explicit risk assessments^25,26^.

In the HCE, studies regarding negative interactions between cetaceans and anthropogenic activities have focused on catches and bycatch off the Peruvian coast^27–29^, and to a lesser extent off Chile and Ecuador^30–33^. Additionally recent small-scale studies are starting to focus on other risk sources and their evaluation, such as ship strikes and the effect of whale-watching boats on cetacean behavior^34–36^.

In 2016, the Instituto de Fomento Pesquero (The Fishery Development Institute, IFOP) started a monitoring program for cetaceans inhabiting the HCE off the Chilean coast. Research cruises that regularly undertake systematic oceanographic and fishery resources monitoring were used as survey platforms. Paralleling these efforts, during 2020 the Servicio Nacional de Pesca y Acuicultura (Chilean National Fisheries and Aquaculture Service, SERNAPESCA) made publicly available daily vessel tracking data for four vessel fleets operating in Chilean waters. Based on these efforts, here we present the first estimates of cetacean distribution and abundance for the Chilean portion of the HCE along with assessment on the spatial overlap between cetaceans and vessel traffic in the area.

## Methods

### Line-transect surveys

**Figure 1:**
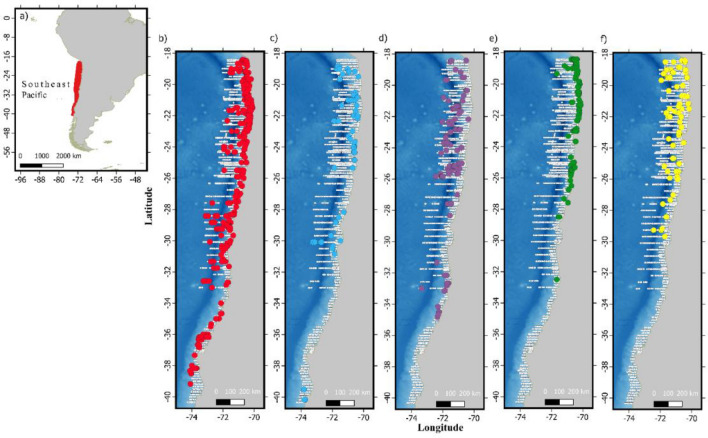
(**a**) Red polygon indicates the location and coverage of the study area in the Southeast Pacific. (**b**) Red points indicate fin whale sightings, (**c**) light-blue points indicate blue whale sightings, (**d**) purple points indicate sperm whale sightings, (**e**) green points indicate dusky dolphin sightings, and (**f**) yellow points indicate common dolphin sightings. Small white dots denote the midpoint for each on-effort track segment. Data layers (including maps) were created in R v. 4.0.2 (www.r-project.org) and ensembled in QGIS v. 3.8.0 (www.qgis.org) for final rendering. Maps were created using data on bedrock topography from the National Centers for Environmental Information (https://maps.ngdc.noaa.gov/viewers/grid-extract/index.html). Grid-cells with values above 0 were considered land coverage and assigned a uniform color.

Line-transect surveys were carried out off coastal waters of the Chilean HCE (18° 00′ S–41° 00′ S) during austral summer, autumn and spring of 2016–2021 (Supplementary Table S1), covering up to ~ 200 km from the nearest coast of Arica in the north and Valparaiso in the central portions of the study area, narrowing to ~ 90 km in the south (Fig. 1). Surveys were carried out across 26 cruises, which covered overlapping subsections within the 407,337 km^2^ study area. Field protocols followed standard line-transect survey methods^37^. Two to three dedicated observers searched for cetaceans using 7 × 50 handheld binoculars. Once a group was detected, radial angle and reticle readings from the binoculars were recorded and then used for calculating the perpendicular distance from the sighting to the transect. Species identification was not possible on all occasions and were recorded to the lowest taxonomic level possible (Table 1). We divided on-effort tracks from line-transect surveys into contiguous 10-km sampling segments^38,39^ and remanent shorter sampling segments of length L (range 0.1–10, quartile 25% = 8.2, 50% = 9.6, 75% = 10). For each one of these sampling segments, the response variable group counts (*n*_*i*_) and environmental covariates were extracted assuming the midpoint of each track as the spatial point from which the covariates were extracted. Data from the first five cruises (2016–2017), encompassing 144 days at sea, were discarded as only one observer was allowed on board during the first year of the program.

**Table 1:** Summary information for all predictors used for modeling cetacean abundance and distribution. The processing column indicates how were the data manipulated after extraction from sources.

Variable	Abbreviation	Units	Spatial resolution	Temporal resolution	Processing	Source
Logarithm of Chlorophyll-*a* concentration	LOGCHL	mgC m-3	4.64 km × 4.64 km	Monthly	3-Month average	Level 3 Aqua MODIS. ERDAP Data set ID: erdMH1chlamday
Logarithm of Chlorophyll-*a* concentration Long-term	CHLLT	mgC m-3	4.64 km × 4.64 km	Static	5-Year average	Based on the above logarithm of Chlorophyll-*a* concentration data
Sea surface temperature	SST	°C	1 km × 1 km	Daily	None	Level 4 multi-scale Ultra-high Resolution. ERDAP Dataset ID: jplMURSST41
Sea surface temperature Long-term	SSTLT	°C	2 km × 1 km	Static	5-Year average	Based on the above sea surface temperature data
Sea surface temperature anomaly	SSTA	°C	0.01° × 0.01°	Daily	None	Level 4 multi-scale Ultra-high Resolution. ERDDAP Dataset ID: jplMURSST41anom1day
Thermal gradient	TG	°C	10 km × 10 km	Daily	Calculated using the R package "grec"^46^ and rescaled to 10 km resolution	Based on the above sea surface temperature data
Depth	DEPTH	Meters	0.01666667° × 0.01666667°	Static	None	ETOPO1 1 arc-minute global relief model of Earth's surface. ERDDAP Data set ID: etopo360
Slope	SLO	Radians	0.01666667° × 0.01666667°	Static	Calculated using the R package raster^48^	Based on the above depth data
Julian day	JD				None	

After removing data from the first year, a total of 3927 sampling segments were included in the analyses, a total of 33,500 km on-effort. Wave height (meters), wind speed (knots) and sea state (Beaufort scale) were recorded whenever effort started or was resumed and were used as observational covariates for modelling detection probability. These covariates were transformed to binary categorical variables. Wave height ≤ 1.75–2.25 m corresponded to 0 and 1 above this threshold. Wind speed ≤ 17–21 knots corresponded to 0 and 1 above this threshold. Sea state ≤ 2 corresponded to 0 and 1 above this threshold. Effort was stopped when sea state was Beaufort 4 or greater.

### Satellite oceanographic and topographic predictors

Environmental predictors were chosen based on previous studies and ecological expectations about the studied cetacean’s species. Fin whales have been associated with productive waters modified by upwelling events, frontal features and steep sea bottom slope^26,40,41^, therefore Chlorophyll-*a*, thermal gradients (TG), sea surface temperature (SST), sea surface temperature anomaly (SSTA) and bathymetric slope (SLO) were used as predictors. Blue whale distribution has been correlated with productivity patterns and frontal features in the adjacent Chilean Northern Patagonia^42,43^, therefore Chlorophyll-*a* and TG were used as predictors. Given known blue whale migratory patterns^19^, the date of each segment was transformed into Julian day (JD) and used as a predictor as well. For sperm whales bathymetric features and SST have been correlated with their presence^44^, therefore SST, DEPTH and SLO were used as predictors. For dusky and common dolphins SST and DEPTH have been correlated with their distribution in the adjacent Peruvian portion of the HCE^20^, therefore here we used the same predictors. In addition, for dusky dolphins we also used SLO given their observed sightings associated to the flat shallow waters of northern Chile. For all species considering SST and Chlorophyll-*a* as predictors alternative models were constructed using time-variant versions and static long-term averages of these variables.

Chlorophyll-*a*, daily SST, daily SSTA, and DEPTH data were extracted using R package “rerddapXtracto” v. 1.1.0^45^, which accesses the ERDDAP server at the NOAA/SWFSC Environmental Research Division (Table 2). For each track, the logarithm Chlorophyll-*a* (LOGCHL) represented the average value for the current and the two previous months since the sampling date. In addition, long-term concentration of Chlorophyll-*a* (CHLLT) and sea surface temperature (SSTLT) were considered as the mean of 2017–2021. From SST maps, TG maps were generated for each day survey data were available using the R package “grec” v. 1.4.1^46^ with the Contextual Median Filter algorithm^47^ as the method for calculating gradients. Original TG maps were rescaled to 10 × 10 km grid size raster, and values were averaged over these new grid-cells. From the DEPTH map, SLO were calculated using the R package raster^48^. Prior to analysis, correlations were assessed using Pearson correlation analysis and all variables were centered and scaled.

**Table 2:** Summary of species recorded during surveys conducted off the HCE. Common dolphins and pilot whales were identified to the genus level; hence sighting could represent either of the two species described for each genus. The truncations distance used for fitting the detection function is provided for each species with at least 30 sightings. The number of available sightings after truncation and removing data from the first five cruises is specified as used sightings.

Species		Total sightings	Truncated distance	Used sightings
Fin whale	*Balaenoptera physalus*	371	2.5	321
Blue whale	*Balaenoptera musculus*	70	2.5	58
Sperm whale	*Physeter macrocephalus*	127	2.5	112
Dusky dolphin	*Lagenorhynchus obscurus*	166	1.5	145
Common dolphins	*Delphinus * spp.	77	2	65
Sei whale	*Balaenoptera borealis*	11	–	–
Humpback whale	*Megaptera novaeangliae*	9	–	–
Unidentified large whale		167	–	–
Orca	*Orcinus orca*	10	–	–
Bottlenose dolphin	*Tursiops truncatus*	25	–	–
Risso's dolphin	*Grampus griseus*	10	–	–
Pilot whales	*Globicephala * spp.	27	–	–
Pigmy killer whale	*Feresa attenuata*	2	–	–
Unidentified delphiniid		22	–	–
Total		1094		

### Modelling approach

Binomial N-mixture models (BNMM) allow the estimation of true counts based on observed counts gathered through imperfect detection^49^. This type of model fully propagate uncertainty between the observation and distribution sub-models and allows the estimation of true counts even when observed data are equal to zero. Based on the BNMM from Ref.^50^ the true number of groups *N*_*i*_ for each track-segment *i* was modelled by a Zero-inflated Poisson distribution

$$Pr\left\{{N}_{i}>0\right\}=\sum\psi \frac{{{\lambda }_{i}}^{{N}_{i}}{e}^{{-\lambda }_{i}}}{{N}_{i}!},$$

where *ψ* is the probability of a non-zero *N*_*i*_ and *λ*_*i*_ is the usual Poisson parameter, which depends on the exponential of a linear function of covariates

$${\lambda }_{i}={2WL}_{i}\times {e}^{({\beta 0}+ {\beta X}_{i}+{\tau }_{i})}.$$

*β*_*0*_ is an intercept, ***β*** is a vector of parameters coefficients, *X*_*i*_ is the corresponding design matrix and τ_i_ is an observation-level random effect to account for overdispersion. *W* corresponds to the truncation distance and *L*_*i*_ the track segment length. The resulting area (*2WL*_*i*_) is an offset term accounting for sampling effort.

A binomial distribution relates the observed number of groups counts *n*_*i*_ to *N*_*i*_ with probability of success determined by detection probability *p*_*i*_, thus making *N*_*i*_ a latent variable.

$$Pr\left\{{n}_{i}|{N}_{i},{p}_{i}\right\}=\left(\genfrac{}{}{0pt}{}{{N}_{i}}{{n}_{i}}\right){{p}_{i}}^{{n}_{i}}{(1-{p}_{i})}^{{N}_{i}-{n}_{i}}.$$

*p*_*i*_ was derived from un-binned perpendicular distances *y*_*d*_ from each _*d*_ detection*,* using a half normal distribution with the parameter *σ*_*i*_ modelled as the function of observational covariates wind speed, wave height and sea state.

$${\sigma }_{d}={e}^{({Y}_{d}\alpha )}.$$

*α* is a vector of parameter coefficients and *Y*_*d*_ is the corresponding design matrix, which in this case are dummy values of categorical variables.

Model fit was assessed through a simulation-based approach and scaled residuals diagnostics using the R package DHARMa v. 0.4.5^51^. Within this package quantile dispersion, outliers and deviation tests were implemented. Additionally, the percentage of zeros in observed and simulated data was inspected. Posterior predictive check was used based on chi-squared tests^52^, which allowed us to calculate the ratio between the sum of discrepancy measures in observed and simulated data, the c-hat parameter, and a Bayesian p-value, which is the probability to obtain a test statistic that is at least as extreme as the observed test statistic computed from the actual data^53^. Residual autocorrelation was assessed through Moran I test based the Euclidian distances between sample coordinates^51^.

Based on these models, spatial predictions of *N* (*N*_*g*_) were estimated for each grid cell *g* in a 7 × 7 km grid holding selected environmental predictors. The expected number of individuals within a grid cell was obtained as the product of mean group size, *N*_*g*_ and grid cell area. Abundance estimates were obtained as the sum of all expected density of individuals in each grid cell. Abundance estimates posterior distributions were obtained by replicating the previous step for all values in parameters posterior distributions. When selected models used time-variant predictors, prediction grids were used for each species considering conditions observed in Jan, Apr, and Oct of each study year, representing summer, autumn and spring conditions, respectively. Seasonal abundance estimates were obtained by pooling posteriors across the same months each year. As large variability is expected to occur depending on the oceanographic conditions used for abundance estimates, overall abundance estimates were obtained by combining all seasonal abundance posteriors. These were intended to reflect our uncertainty on the suitability of specific seasonal conditions.

All models were fit in R v. 4.0.2 3^54^ and JAGS v. 4.3.0^55^ for Markov Chain Monte Carlo estimation methods. Vague priors were used for all parameters. Three chains were run in parallel through 70,000 iterations each. The first 40,000 samples were discarded as burn-in, and 1 out of every 50 remaining samples was retained, for a total of 1800 samples to form the posterior distribution of model parameter estimates.

### Defining spatial overlap with marine traffic

To characterize vessel traffic patterns in the area, daily vessel tracking information (time-stamped GPS locations for individualized vessels) was obtained from SERNAPESCA, available at www.sernapesca.cl. Details about this database have been summarized elsewhere^43^. Briefly, vessel data involving the industrial and artisanal fisheries, aquaculture, and transport fleets were analyzed. Artisanal fishing fleet comprises vessels up to 18 m in length and less than 80 cubic meters of storage capacity; above these metrics fishing vessels are considered part of the industrial fishing fleet. The transport fleet comprises vessels with no size limitations, engaged solely in the transportation of fishery resources. The aquaculture fleet is the most diverse one considering its different operations (e.g., transport of personnel, live and processed animals, and supplies, and infrastructure movement) with vessel sizes ranging from 5 to 100 m. All procedures described next were conducted independently for each fleet during data analyses. We used a 7 × 7 km grid to calculate vessel density (*VD*_*i*_) for each grid-cell *i*. Vessel data are provided daily, with data gaps occurring for some days. Therefore, *VD*_*i*_ was calculated by summing the daily number of unique vessels crossing each grid-cell *i* in a month divided by the total number of days with available data (range 25–31 days). This procedure was conducted for austral summer, autumn and spring months (Jan–Jun and Oct–Dec of each year) and then averaged into a single layer. Data from austral winter months were not used because no surveys were conducted during this season.

A quantitative measure of risk associated to vessel traffic can be considered as a monotonic function of the number of vessels and the probability of encountering cetaceans^25,56^. Therefore, as a measure of risk we calculated the relative probability of cetacean-vessel encounter (RPCVE)^25,57^ by combining the predicted number of cetacean groups in a grid cell (N_*i*_), and VD_*i*_ as follows:

$${RPCVE}_{i}=\frac{{Pw}_{i} { Pv}_{i}}{\sum_{i=1}^{n}{(Pw}_{i} { Pv}_{i})}$$

where ${Pw}_{i}=\frac{{N}_{i}}{\sum_{i=1}^{n}({N}_{i})}$ corresponds to the probability of observing a cetacean within each grid-cell *i* relative to all other grid cells *n*, and ${Pv}_{i}=\frac{{VD}_{i}}{\sum_{i=1}^{n}{(VD}_{i})}$ corresponds to the observed number of vessels within grid-cell *i* relative to all other grid cells *n*. Finally, to generate quantitative estimates on the degree of overlap between cetacean distribution and vessel traffic we used the Shoener's D and Warren's I similarity statistics^58^. These statistics range from 0 indicating no overlap, to 1 indicating that distributions are identical. To use these statistics, the variables N_*i*_ and VD_i_ were rescaled to range between 0 and 1 and inputted to the *nicheOverlap* function from the R package *dismo*^26,59^.

## Results

A total of 12 species or group of species were recorded during the on-effort sampling segments (Table 2). Of the baleen whales, only fin (*Balaenoptera physalus*) and blue whale (*Balaenoptera musculus*) had sufficient sightings for fitting the detection function, with the former representing the most common species observed in the entire study area. The most common odontocetes observed were dusky dolphins (*Lagenorhynchus obscurus*), sperm whales (*Physeter macrocephalus*) and common dolphins (*Delphinus* spp.) with the latter only identified to the genus species. These five species (or genus) were selected for modelling.

Among the three observational covariates, wave height showed an important effect on detection probability, except for common dolphins where no observational covariate affected detectability (Supplementary Figs. S1–S5). Table 3 summarizes estimated group size parameters and abundance for each cetacean species.

**Table 3:** Mean, standard deviation, median and credible intervals for group size parameters and abundance estimates.

Species	Mean	SD	2.50%	Median	97.50%
**Group size mean**					
Fin whale	2.0	0.1	1.9	2.0	2.2
Blue whale	1.7	0.2	1.4	1.7	2.0
Sperm whale	2.7	0.2	2.4	2.7	3.0
Dusky dolphin	18.6	1.3	16.3	18.5	21.2
Common dolphin	46.9	5.6	37.8	46.4	58.6
**Group size variance**					
Fin whale	2.2	0.1	2.0	2.2	2.5
Blue whale	1.8	0.2	1.4	1.7	2.2
Sperm whale	3.0	0.3	2.5	3.0	3.8
Dusky dolphin	272.7	46.7	199.9	268.1	377.3
Common dolphin	2214.3	667.3	1274.2	2094.5	3776.5
**Abundance**					
Fin whale	2511.6	671.0	1387.9	2383.8	5068.5
Blue whale	923.7	461.7	278.1	817.6	2998.2
Sperm whale	2236.5	539.3	1289.9	2162.7	4633.2
Dusky dolphin	7509.2	4285.2	3914.2	7032.3	17,633.7
Common dolphin	16,812.1	5556.2	8412.5	15,813.1	38,194.9

Unless specifically indicated, dispersion, outliers and deviation tests, percentage of zeros in simulated data, Bayesian *p*-values and c-hat showed good model fitting in all selected models (Supplementary Tables S2–S13, Supplementary Figs. S6–S17). For all species distance-based autocorrelation was never statistically significant (Moran's I test, *p* > 0.05).

### Fin whale

**Figure 2:**
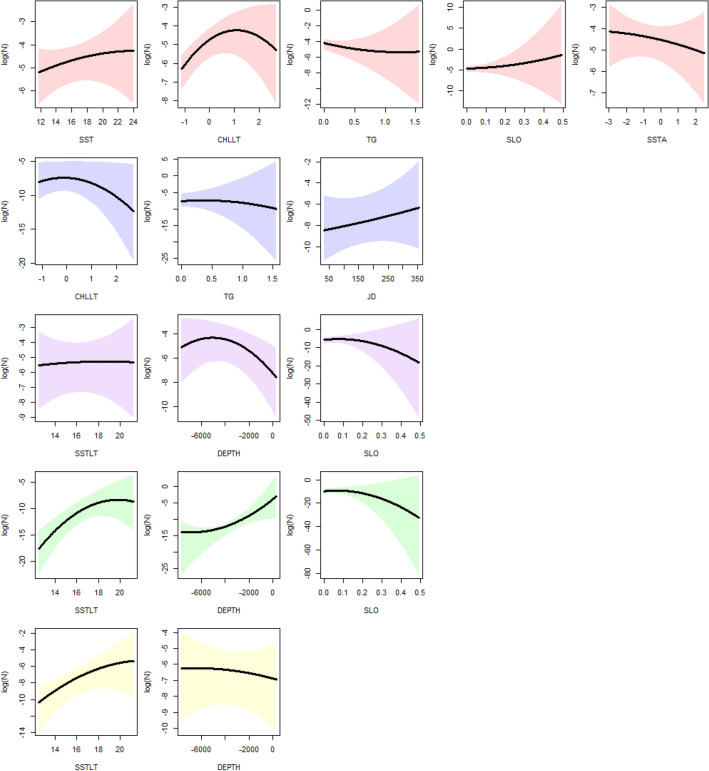
From top to bottom, rows show the predicted response (number of groups in log scale) to environmental predictors for (**a**) fin whale (red), (**b**) blue whale (blue), (**c**) sperm whale (purple), (**d**) dusky dolphin (green), and (**e**) common dolphins (yellow). Black thick lines indicate median responses and colored shaded areas the credible intervals. Predictors used are, sea surface temperature (SST), long-term average in sea surface temperature (SSTLT), sea surface temperature anomaly (SSTA), long term average in Chlorophyll-*a* concentration in log-scale (CHLLT), thermal gradients (TG), depth (DEPTH), slope (SLO) and Julian day (JD).

**Figure 3:**
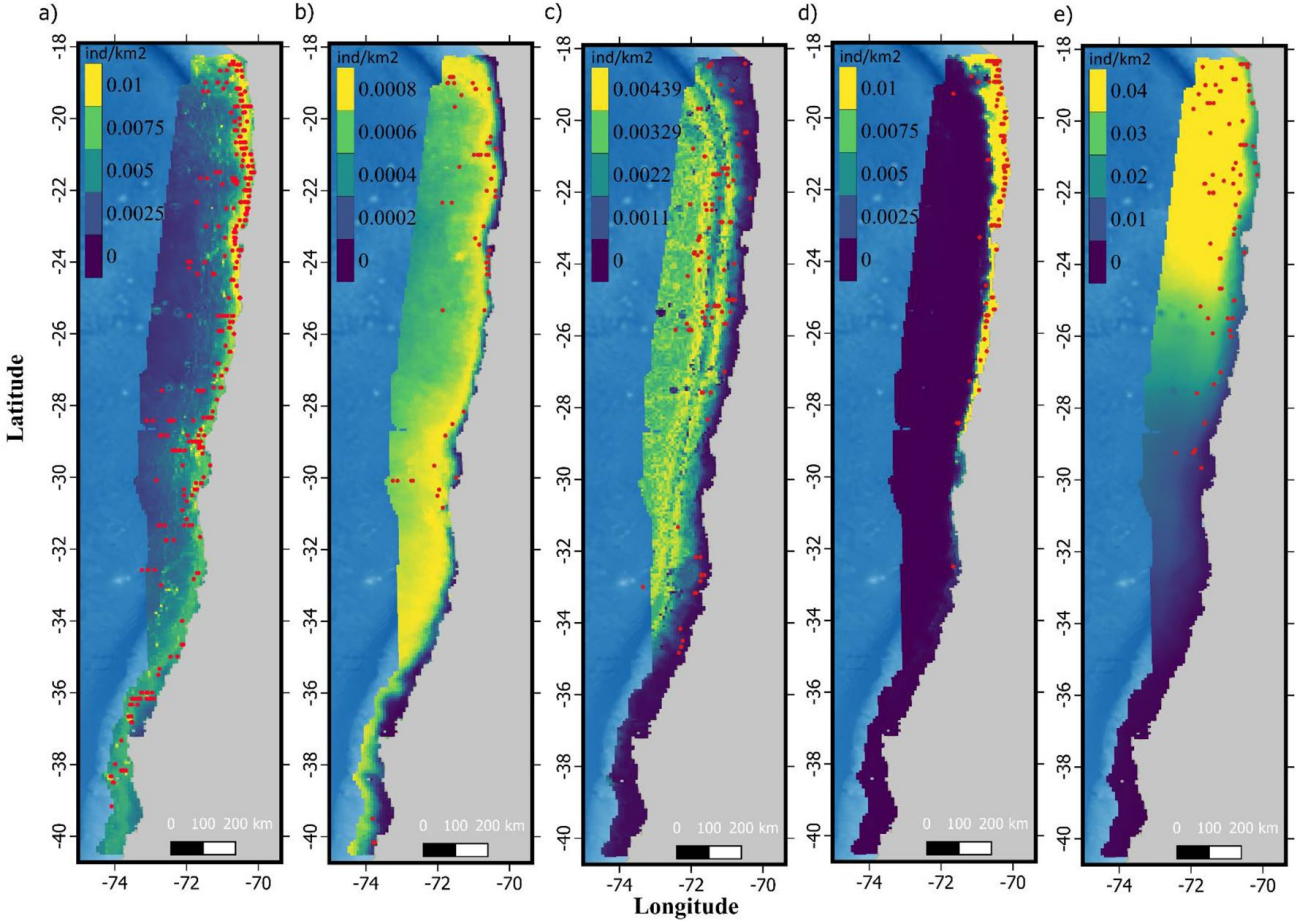
Predicted distribution for (**a**) fin whale, (**b**) blue whale, (**c**) sperm whale, (**d**) dusky dolphin and (**e**) common dolphins considering results from the Binomial N-mixture model. Data layers (including maps) were created in R ver. 4.0.2 (www.r-project.org) and ensembled in QGIS ver. 3.8.0 (www.qgis.org) for final rendering. Maps were created using data on bedrock topography from the National Centers for Environmental Information (https://maps.ngdc.noaa.gov/viewers/grid-extract/index.html). Grid-cells with values above 0 were considered land coverage and assigned a uniform color.

For fin whales 4 models were compared considering all possible combinations of using dynamic and long-term averages of Chlorophyll and sea surface temperature predictors. In all cases some quantile deviation was observed (Supplementary Figs. S6–S9), however, the model considering CHLLT, and SST reduced this effect to the minimum (Supplementary Fig. S8). Model results showed that fin whale densities presented a positive correlation with SST, mid to high values of CHLLT and a negative one with TG and SSTA (Fig. 2). Spatial predictions of fin whale distribution showed a general pattern of higher densities closer to the coast, especially in the northern part (Fig. 3a).

### Blue whales

For blue whales models using LOGCHL and CHLLT yielded no appreciable differences (Supplementary Tables S6–S7 and Supplementary Figs. S10–S11). The model including CHLLT was selected for easiness. Blue whale densities were expected to be higher at low- to mid-CHLLT and high-JD (Fig. 2). Spatial distribution for blue whales showed a preference for areas away from the coast with lower densities expected for the southern portion of the study area (Fig. 3b).

### Sperm whales

For sperm whales models using SST and SSTLT yielded no appreciable differences (Supplementary Tables S8–S9 and Supplementary Figs. S12–S13), but the one using SSTLT agreed more with observed data when considering spatial predictions. Sperm whale densities were expected to peck around 5000 m depth (Fig. 2). Spatial predictions indicated higher densities in offshore waters of the central and northern portions of the study area (Fig. 3c).

### Dusky dolphin

For dusky dolphins the model using SSTLT provided with a better fitting than the one using SST, which presented significant deviation in residual quantiles (Supplementary Tables S10–S11 and Supplementary Figs. S14–S15). Dusky dolphin counts were positively correlated with SSTLT and DEPTH (higher values are less negative, thus shallower waters, Fig. 2). Dusky dolphin showed the most restricted distribution of all species, distributed mostly in coastal waters in the northern and central portions of the study area (Fig. 3d).

### Common dolphin

For common dolphins the model using SSTLT provided with a better fitting than the one using SST, which presented a small non-significant deviation in residual quantiles (Supplementary Tables S12–S13 and Supplementary Fig. S16–S17). In addition, the selected model agreed more with observed data when considering spatial predictions. Common dolphins’ counts were positively correlated with SSTLT (Fig. 2). Spatial predictions for common dolphins showed a preference for coastal and offshore waters of the northern portion of the study area (Fig. 3e).

### Overlap with vessels

**Figure 4:**
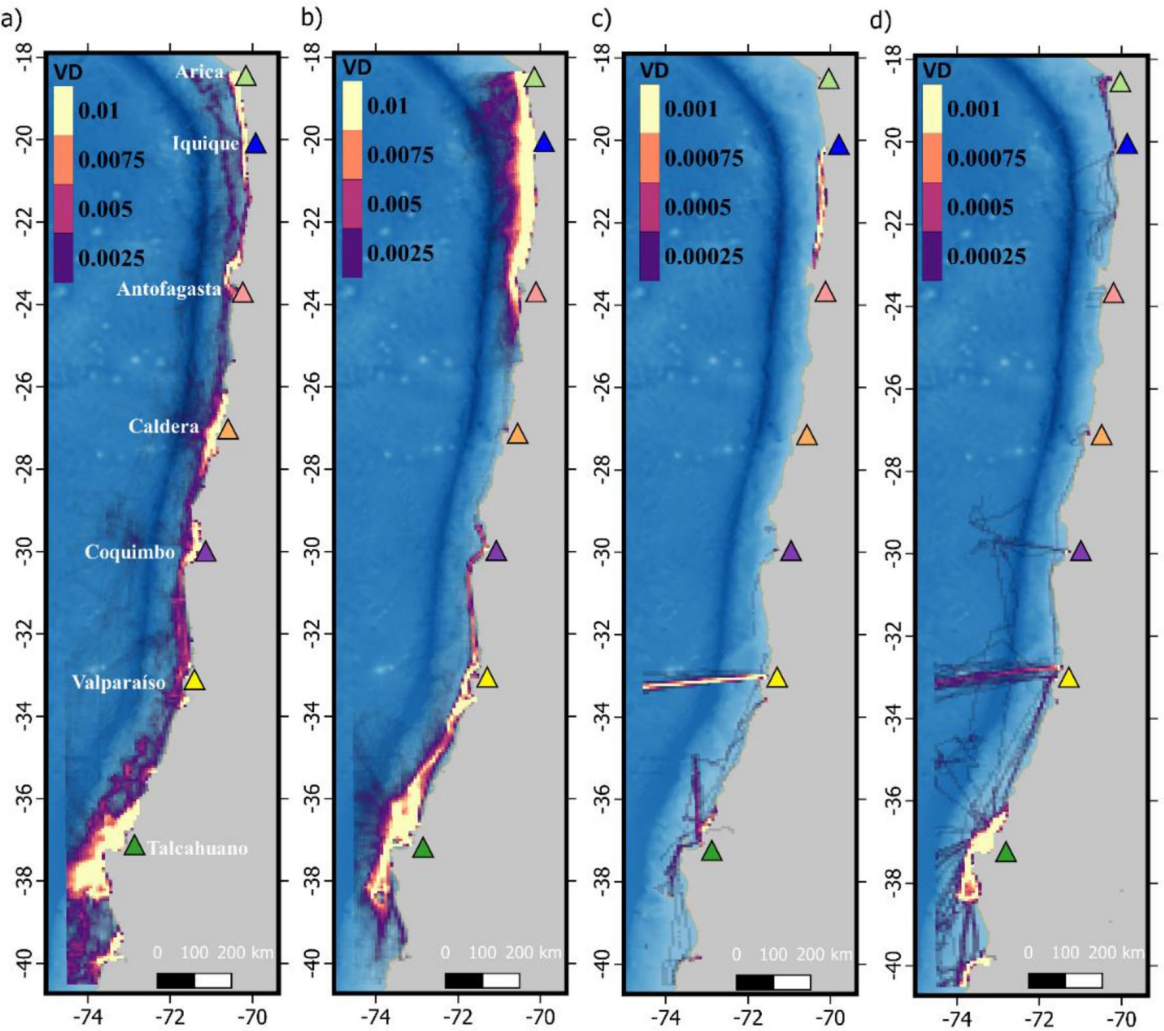
Vessel density (VD) as the mean number of vessels per km^2^ per day, for the artisanal fishery (**a**), industrial fishery (**b**), transport (**c**) and aquaculture (**d**) fleets. Colored triangles indicate the location of most important ports. Data layers (including maps) were created in R ver. 4.0.2 (www.r-project.org) and ensembled in QGIS ver. 3.8.0 (www.qgis.org) for final rendering. Maps were created using data on bedrock topography from the National Centers for Environmental Information (https://maps.ngdc.noaa.gov/viewers/grid-extract/index.html). Grid-cells with values above 0 were considered land coverage and assigned a uniform color.

**Figure 5:**
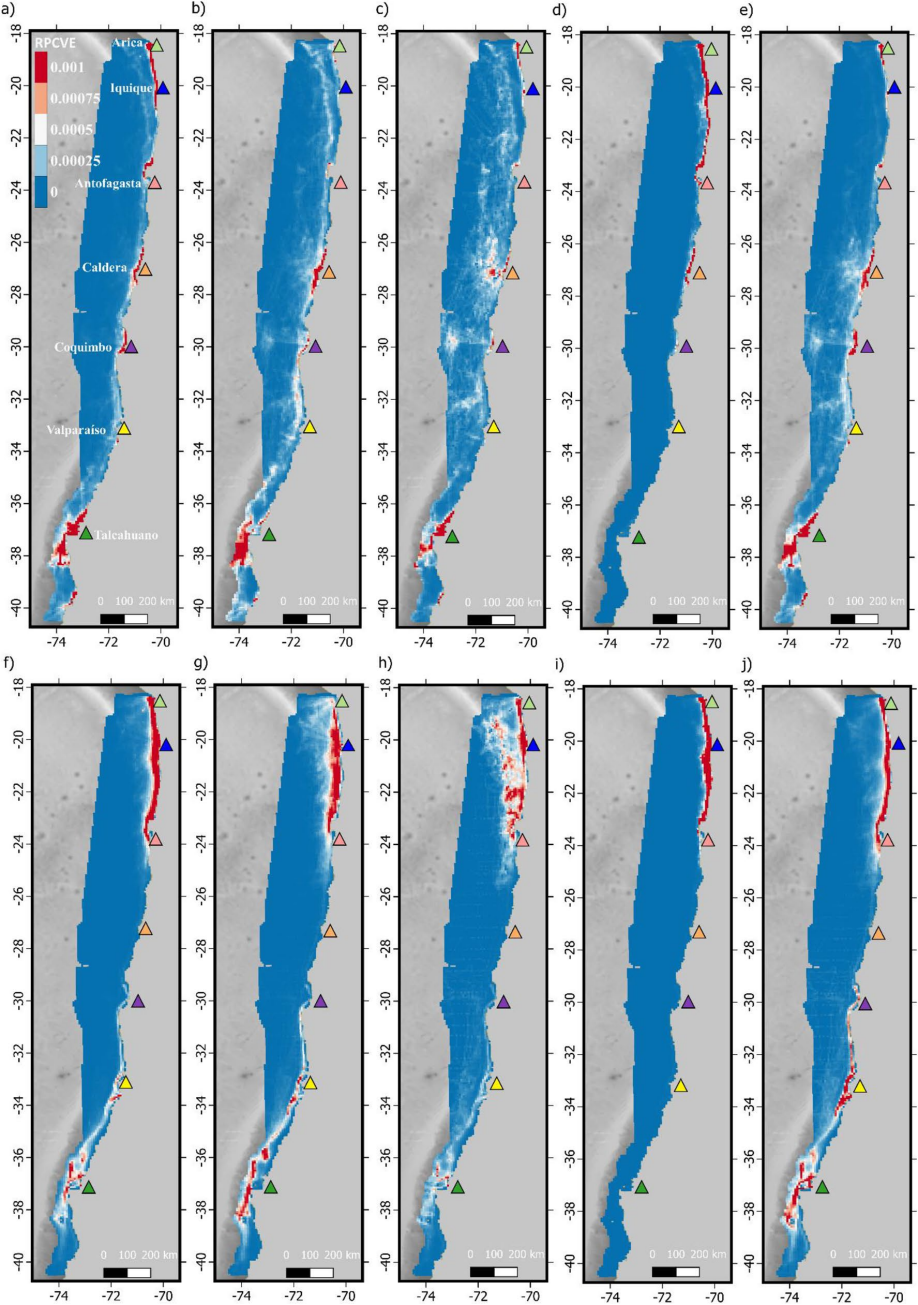
Predicted relative probability of cetacean-vessel encounter (RPCVE) for artisanal fishery fleet (**a**–**e**) and industrial fishery fleet (**f**–**j**). Columns show results for fin whales (**a**,**f**), blue whales (**b**,**g**), sperm whales (**c**,**h**), dusky dolphins (**d**,**i**) and common dolphins (**e**,**j**). Results for transport and aquaculture fleets are provided in Supplementary Fig. S18. Colored triangles indicate the location of most important ports. Data layers (including maps) were created in R ver. 4.0.2 (www.r-project.org) and ensembled in QGIS ver. 3.8.0 (www.qgis.org) for final rendering. Maps were created using data on bedrock topography from the National Centers for Environmental Information (https://maps.ngdc.noaa.gov/viewers/grid-extract/index.html). Grid-cells with values above 0 were considered land coverage and assigned a uniform color.

VD absolute values were highest for the artisanal fleet (range 0–41.3) followed by industrial fishery (0–22.6), aquaculture (range 0–1.6) and transport (range 0–0.3) fleets. Figure 4 summarizes VD values expressed in mean number of vessels per day per km^2^. In general, RPCVE maps showed that irrespectively of the fleet or species considered, two main areas located at the northern and southern portions of the study area hold the highest relative probability of cetaceans interacting with vessels (Fig. 5).

The aquaculture and transport fleets showed considerably lower overlap scores than both types of fishing fleets (Table 4). The dusky dolphin presented the highest overlap scores with the industrial fishery fleet (D = 0.487 and I = 0.702) exceeding those found for any other fleet or species (Table 4). Fin whale showed the second highest overlap index with the industrial fishery fleet (D = 0.421 and I = 0.699) and the highest one with the artisanal fishing fleet (D = 0.355 and I = 0.638).

**Table 4:** Shoener's D and Warren's I similarity statistics for each cetacean species and vessel fleet type.

	Fin whale		Blue whale		Sperm whale		Dusky dolphin		Common dolphins	
	D	I	D	I	D	I	D	I	D	I
Aquaculture	0.192	0.344	0.123	0.216	0.065	0.143	0.047	0.154	0.104	0.216
Artisanal	0.355	0.638	0.232	0.463	0.132	0.352	0.209	0.424	0.192	0.433
Industrial	0.421	0.699	0.231	0.452	0.120	0.300	0.487	0.702	0.113	0.304
Transport	0.140	0.306	0.053	0.162	0.020	0.101	0.259	0.379	0.045	0.157

## Discussion

Most of the species analyzed here were concentrated in the northern portion (~ 18° S–30° S) of the study area (Figs. 1 and 3). This area is characterized by persistent upwelling events that are less seasonally modulated than the southern portion, which experiences a major decrease in primary productivity during the austral winter^60,61^. The five cetacean species, however, presented highly variable preferences in the dynamic habitat characteristics selected.

Fin whale was the most common species found in the HCE (n = 371). Although more frequent in the central and northern portions (Figs. 1, 3), this was the only species present throughout the entire study area. Studies off Northern Chile, indicate that the dominant euphausiid species *Euphausia mucronata*^7,62^ is the main prey for fin whales^18,63^. *E. mucronata* is known to feed largely on diatoms in the austral spring and summer^64^ and is associated with areas adjacent to upwelling centers in the HCE^65,66^. This corresponds with our results where fin whales were associated to high SST, reflecting their preference for the northern portion of the study area, and low values of SSTA and mid- to high-CHLLT (Fig. 2, Supplementary Table S4), typical of waters modified by coastal upwelling. Given fin whale estimated daily average consumption of ~ 8 tons of krill^67^, our abundance estimate (median 2383.8, SD 671, CI 1387.9–5068.5) indicate that this sole species might remove ~ 7 × 10^6^ tons of krill a year. Considering that the role of large cetaceans in the ecosystem functioning and biogeochemical cycling of the HCE has been traditionally undermined^1,4,7,61^ our results highlight the importance that fin and other baleen whales might elicit in HCE structuring^68–71^.

Blue whales were far less common than fin whales (n = 70). This was expected as currently most of southeast Pacific population concentrate in Northern Chilean Patagonia, during the austral summer and autumn^19,42,43,72^ where they prey upon patches dominated by the euphausiid species *E. vallentini*^73^. Blue whales are occasionally sighted off the HCE but deemed rare^22,74^, with few observations of them preying upon *E. mucronata*^18^. Blue whale counts were negatively correlated with CHLLT (Fig. 2, Supplementary Table S6). Because this is the opposite to what has been recorded for this and other blue whale populations^43,75,76^ and that 47 out of 58 sightings were observed during spring months (a peak also shown by JD, Fig. 2), results suggest that most blue whales sighted in the HCE might be individuals migrating towards their primary summering ground off Chilean Northern Patagonia^19,42,43,72^. Although previous abundance estimates for blue whales in the southeast Pacific are not directly comparable in terms of covered areas and time periods, estimates from this study lay within the range of previous estimates^22,42,77^.

In the oceanographically analogous California Current Ecosystem, fin and blue whales can occur sympatrically, sharing common euphausiid resources^78,79^. Based on twentieth century whaling data this was also the case for the HCE where thousands of blue and fin whales were caught along the coast of Chile, Peru, and Ecuador, with most catches in Chile concentrating within our study area^75,80–82^. The question remains about why, off the Chilean coast and almost 40 years after whaling operations ceased, blue whales are still scarce outside the Northern Patagonia, suggesting their population may be far from recovery.

Sperm whales were the third most common cetacean species (n = 127) recorded off the HCE (Table 2). A previous study by Ref.^83^ examined sperm whale habitat in the HCE off Northern Chile and found that sperm whales were adjacently associated with two upwelling centers off Northern Chile (latitudes ~ 18° S and ~ 23° S). Our results confirm the association of sperm whale with the northern part of the study area and a preference for mid- to high-DEPTH (Fig. 2, Supplementary Table S8). Given that sperm whales are known to feed off mesopelagic squid a trophic lag might explain the spatial mismatch between areas of high primary productive and high sperm whale density^83,84^. Mediated by advected water (plumes) generated by nearby upwelling centers high secondary productivity might secure prey resources for sperm whale offshore^76,85^. This is congruent with results of the alternative predictor set used for modelling sperm whale counts, which showed a negative correlation with SST (Supplementary Table S9). Although spatial predictions based on SST monthly conditions predicted higher densities in the colder southern portion of the study area, which was incongruent with the data, the fact that daily SST was an important predictor for sperm whales shows that highly dynamic oceanographic features are important for these species.

The dusky (n = 166) and the common dolphin (n = 77) were the two most recorded delphinids, with the former being the second species with most sightings (Table 1). The dusky dolphin also presented the most restricted distribution, concentrated in shallow waters with high SSTLT in the northern and central portions of the study area (Figs. 1, 2, 3, Supplementary Table S10). This corresponds with the reported distribution of the species in Peruvian waters^20^. The better fitting obtained by using SSTLT rather than SST indicates dusky dolphins prefer consistently warmer waters associated to regular upwelling events in the north in opposition to the highly seasonal upwelling patterns of the southern portion. Evidence indicates that dusky dolphins are opportunistic predators consuming a large range of prey items, particularly pelagic schooling fish and squid^86–90^, but also mesopelagic species typically present in deeper waters^91,92^. Although no description for dusky dolphin diet is available for Chile, the described dietary preferences and distribution described for the species in Peru are coherent with our findings, suggesting that similar patterns may occur at least for Northern Chile. Even though dusky dolphins should be present along the entire Chilean coast, Ref.^93^ proposed the existence of a distributional gap between 36° 30′ S and 46° S and hypothesized that this gap might separate the Peru–Chile population from the Southwest Atlantic population. Although our findings agree with the^93^ hypothesis, reports of a dusky dolphin stranded at 38° 54′ S (Bedriñana-Romano, pers. obs.) suggest the area might be at least sporadically used by this species.

Common dolphins were recorded mainly in the northern zone, where the highest densities occurred in areas of mid to high SST (Fig. 2, Supplementary Table S13). SST is an important covariable, aspects also reported in other studies for this species^94,95^. In fact^95^ highlights that the distribution and abundance of common dolphins is influenced by SST, and observations during La Niña and El Niño events indicate that changes can occur in the abundance patterns of their key prey. Analysis of stomach contents derived from captured and stranded animals indicates that common dolphins are generalist predators with high foraging plasticity because of their opportunistic feeding on abundant species of small epi- and meso-pelagic fish or cephalopods^87,89,96,97^. It should be noted, however, that in this study common dolphin species were considered together under the genus *Delphinus*. Previous studies carried out in the Peruvian zone of the HCE indicate differences in the distribution patterns of common long-beaked (*D. capensis*) and short-beaked dolphins (*D. delphis*), the former having more coastal habits and the latter having more oceanic habits^20^. For this reason, further work should focus on identifying these dolphins at the species level.

Abundance estimates presented here (Table 3) represent the first attempt to provide needed information for cetaceans off the HCE, however they should be interpreted with caution. None of the species´ distributional range was completely covered leading to the quantification of only an undetermined portion of their population size. This was particularly aggravated in the case of baleen whales due to the large number of unidentified animals (Table 2). Additionally, our abundance estimate for sperm whale might be negatively biased considering that visual sightings alone underestimate their presence due to long dive times^98^. Abundance estimates for common dolphins, and possibly for dusky dolphins, might be positively biased as these species are known to be attracted by vessels leading to abundance overestimation^99^. In fact, the histogram on perpendicular distances for common dolphin shows sings of the phenomenon spiking at distances close to zero (Supplementary Fig. S19). Finally, in cases when dynamic predictors are used, abundance estimates will vary depending on the specific environmental conditions used. This, however, is accounted for within our analytical approach. Pooling abundance posterior distributions based on distinct environmental conditions provide a straightforward manner to integrate variability from environmental conditions with fully propagated parameter uncertainty derived from single-step modelling^100^, thus providing a more complete quantification of the uncertainty around abundance estimates ^42,101^.

Overlap between cetaceans´ predicted distribution and vessel traffic was fleet dependent. Among the vessel fleets considered here, artisanal, and industrial fishery fleets presented considerably larger VD values than the aquaculture and transport fleets (Fig. 4). This was expected given that the aquaculture industry in Chile is concentrated in Chilean Patagonia (south of 40° S) and the transport fleet in the available database excludes different types of transport ships like international cargo and tankers, cruise liners, and recreational vessels^43^. The spatial distribution of RPCVE indicates that there is higher risk for cetaceans interacting with vessels in the northern and southern edges of the study area (Fig. 5). This is the area between the ports of Arica and Antofagasta in the north and around the port of Talcahuano in the south (Fig. 5). RPCVE is a function of both VD and expected cetacean densities, therefore higher values can be expected for several configurations of these variables. For the northern area higher RPCVE values were observed for the transport, industrial and artisanal fishery fleets given that both VD and expected cetacean densities were high (Fig. 5 and Supplementary Fig. S18). For the southern area, the disproportionally higher VD values inflate RPCVE values for all fleets, despite the lower expected cetacean densities (Fig. 5 and Supplementary Fig. S18). Dusky dolphins stand from this pattern given their restricted distribution yielding high RPCVE values only in the northern and central areas for both fishing fleets (Fig. 5).

Shoener's D and Warren's I similarity statistics provide information on the degree of overlap between vessels and cetacean distribution, indicating that both artisanal and industrial fishery fleets present the highest overlap with all species, particularly with dusky dolphins and fin whales (Table 4). Comparing results with other studies using the same overlap statistics, indicate that for most species overlap indexes lie within the same range as those reported for blue whales in Northern Chilean Patagonia^43^ and below those observed in the Mediterranean Sea^26^, which is a high intensity vessel traffic area^102^. Results for dusky dolphin and fin whales, however, are similar to those reported in the Mediterranean study, highlighting the higher risk that these two species are subject to.

The detrimental effects of interactions between cetaceans and vessel-based activities are diverse and involve collisions, behavioral disruption, noise exposure, entanglement in fishing gear and prey depletion by fisheries^103–107^. Some of these negative impacts are being evaluated in the HCE, but studies are few and localized^34–36^. In the particular case of bycatch, information for Chilean waters is extremely scarce, sparse over time and in some cases not necessarily confirmed^33,105,108,109^. However, for common and dusky dolphin, among other cetaceans, recorded catches match the areas where high RPCVE values were observed in the northern and central portions of our study area^31,33,108,109^. Indeed, dusky and common dolphins prey items are often the target of commercial fisheries, a reason for why they are frequently bycaught threatening several of their populations^110–113^.

Overall, our results provide the first distribution and abundance estimations for cetaceans in the entire Chilean portion of the HCE. In addition, a spatially explicit risk assessment of cetacean-vessel encounter has been provided, highlighting special concerns about potential negative interactions between fishing vessels and cetaceans. This information is crucial for Chilean fishing industry given the requirement to comply with the U.S. Marine Mammal Protection Act Import Provisions Rule, which demands bycatch standards comparable to those of the U.S. Our results are therefore critical for orienting further assessments on the unquantified negative interactions between cetaceans and vessels in the HCE.

## Supplementary Information

Supplementary Information 1.

Supplementary Information 2.

Supplementary Information 3.

## Data Availability

BUGS code for fitting the BNMM model, raw line transect data and sightings data are available as Supplementary Information.
