# Supplementary material for: Abundance and distribution patterns of cetaceans and their overlap with vessel traffic in the Humboldt Current Ecosystem, Chile
Source: Sci Rep. 2022 Jun 23;12:10639. doi: 10.1038/s41598-022-14465-7 (PMC9226171; doi:10.1038/s41598-022-14465-7)
Supplement: Supplementary file 3 — Supplementary Information 3. [file 41598_2022_14465_MOESM3_ESM.docx]

**Supplementary files**

**Abundance and distribution patterns of cetaceans and their overlap with vessel traffic in the Humboldt Current Ecosystem, Chile**

^*^Luis Bedriñana-Romano^1,2,3^, Patricia Zarate^4^, Rodrigo Hucke-Gaete^1,2,3^, Francisco A., Viddi^1,2^, Susannah J. Buchan^3,5,6^, Ilia Cari^4^, Ljubitza Clavijo^4^, Robert Bello^4^, Alexandre N. Zerbini^7,8,9^

^1^Instituto de Ciencias Marinas y Limnológicas, Facultad de Ciencias, Universidad Austral de Chile, Valdivia, Chile

^2^ NGO Centro Ballena Azul, 5090000, Valdivia, Chile

^3^Centro de Investigación Oceanográfica COPAS Coastal, Universidad de Concepción, 4070043, Concepción, Región del Bio Bio, Chile

^4^Instituto de Fomento Pesquero, Almirante Manuel Blanco Encalada 839, Valparaíso, Chile

^5^Centro de investigación Oceanográfica COPAS Sur-Austral, Universidad de Concepción, 4070043, Concepción, Región del Bio, Chile

^6^Centro de Estudios Avanzados en Zonas Áridas, Raúl Bitran 1305, La Serena, 1700000, Región del Coquimbo, Chile

^7^Cooperative Institute for Climate, Ocean and Ecosystem Studies, University of Washington & Marine Mammal Laboratory Alaska Fisheries Science Center/NOAA 7600 Sand Point Way NE, Seattle, WA, USA

^8^Marine Ecology and Telemetry Research, 2468 Camp McKenzie Tr NW, Seabeck, WA, 98380, USA

^9^Instituto Aqualie, Av. Dr. Paulo Japiassú Coelho, 714, Sala 206, 36033-310, Juiz de Fora, MG, Brazil.

*Corresponding author: [luis.bedrinana.romano@gmail.com](mailto:luis.bedrinana.romano@gmail.com)

Table S1. Summary of cruises´ information used as platforms during line transect surveys.

| **Cruise ID** | **Year** | **Ship** | **Cruise program** | **Months** | **Latitudinal range** | **Cruise Leader** |
| --- | --- | --- | --- | --- | --- | --- |
| CRC_06 | 2017 | ABATE MOLINA | MOBIO MPH | September-October | 18°50`S - 26°00`S | H.REYES J.ANGULO |
| CRC_07 | 2017 | ABATE MOLINA | RECLAN I y II | December | 18°50`S - 25°00`S | FRANCISCO LEIVA |
| CRC_08 | 2018 | ABATE MOLINA | RECLAN III y IV | February | 25°00`S - 31°00`S | FRANCISCO LEIVA |
| CRC_09 | 2018 | ABATE MOLINA | JUREL NORTE | April | 18°50´S - 23°25´S | JOSE CORDOVA |
| CRC_10 | 2018 | ABATE MOLINA | RECLAS V a X | May | 32°00`S - 40°30`S | Alvaro Saavedra |
| CRC_11 | 2018 | ABATE MOLINA | MOBIO MPH | September-October | 18°50`S - 26°00`S | HERNAN REYES |
| CRC_12 | 2018 | ABATE MOLINA | RECLAN I, XV y II | November-December | 18°50`S - 25°00`S | Francisco Leiva |
| CRC_14 | 2019 | ABATE MOLINA | JUREL NORTE | March-April | 18°50`S - 33°00`S | José Cordova |
| CRC_16 | 2019 | ABATE MOLINA | MOBIO MPH | September-October | 18°50`S - 26°03 S | Jessica Bonicelli |
| CRC_13 | 2019 | ABATE MOLINA | RECLAN III y IV | February-March | 25°00`S - 32°00`S | Francisco Leiva |
| CRC_15 | 2019 | ABATE MOLINA | RECLAS V a X | May | 32°00`S - 40°20`S | Alvaro Saavedra |
| CRC_17 | 2019 | ABATE MOLINA | RECLAN I, XV y II | November-December | 18°50`S - 25°00`S | Francisco Leiva |
| CRC_18 | 2020 | ABATE MOLINA | RECLAN III y IV | February-March | 25°00`S - 32°00`S | Francisco Leiva |
| CRC_19 | 2020 | ABATE MOLINA | JUREL NORTE | March-April | 18°50`S - 33°00`S | José Cordova |
| CRC_20 | 2020 | ABATE MOLINA | RECLAS V a X | May-June | 32°00`S - 40°30`S | Alvaro Saavedra |
| CRC_21 | 2020 | ABATE MOLINA | MOBIO MPH | September-October | 18°50´S - 26°00´S | Hernan Reyes |
| CRC_22 | 2020 | ABATE MOLINA | RECLAN I, XV y II | November-December | 18°50´S - 25°00´S | Francisco Leiva |
| CRC_23 | 2021 | ABATE MOLINA | RECLAN III y IV | February-March | 25°00`S - 32°00`S | Francisco Leiva |
| CRC_24 | 2021 | ABATE MOLINA | JUREL NORTE | March-April | 18°50´S - 23°25´S | José Cordova |
| CRC_25 | 2021 | ABATE MOLINA | RECLAS V a X | May-June | 32°00`S - 40°20`S | Alvaro Saavedra |
| CRC_26 | 2021 | ABATE MOLINA | MOBIO MPH | September-October | 18°50`S - 26°00`S | Hernan Reyes |


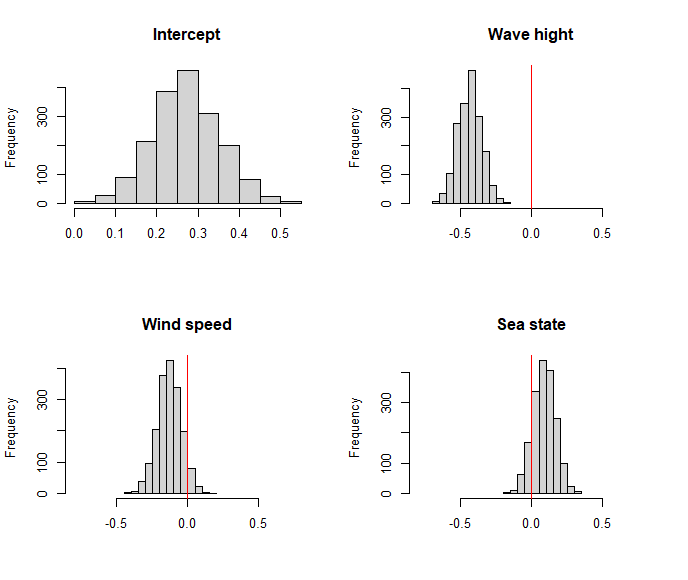


**Figure S1.** Posterior distributions for parameters controlling the effect of observational covariates on detection function for fin whale. Red vertical lines denote zero.


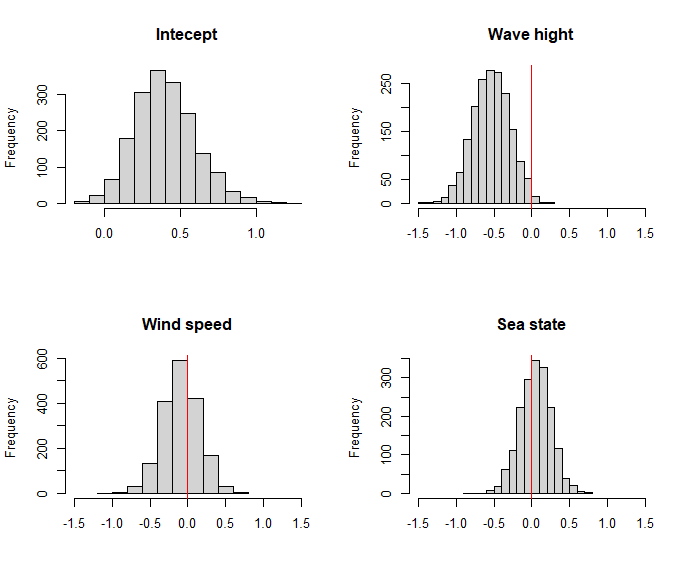


**Figure S2.** Posterior distributions for parameters controlling the effect of observational covariates on detection function for blue whale. Red vertical lines denote zero.


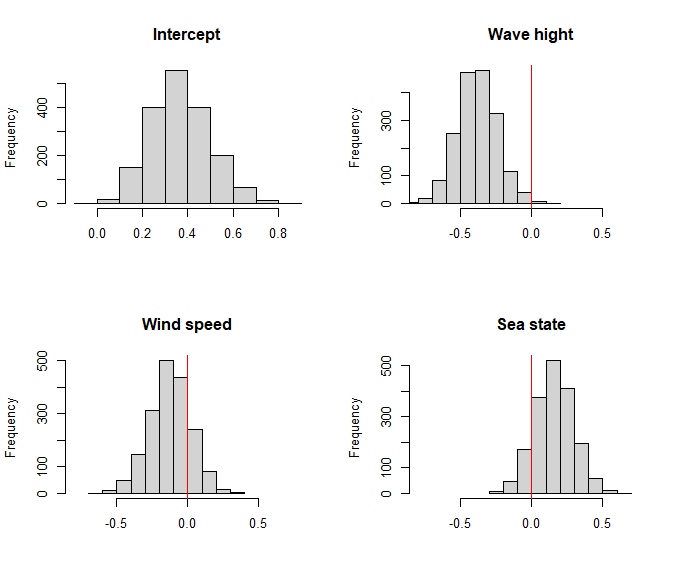


**Figure S3.** Posterior distributions for parameters controlling the effect of observational covariates on detection function for sperm whale. Red vertical lines denote zero.


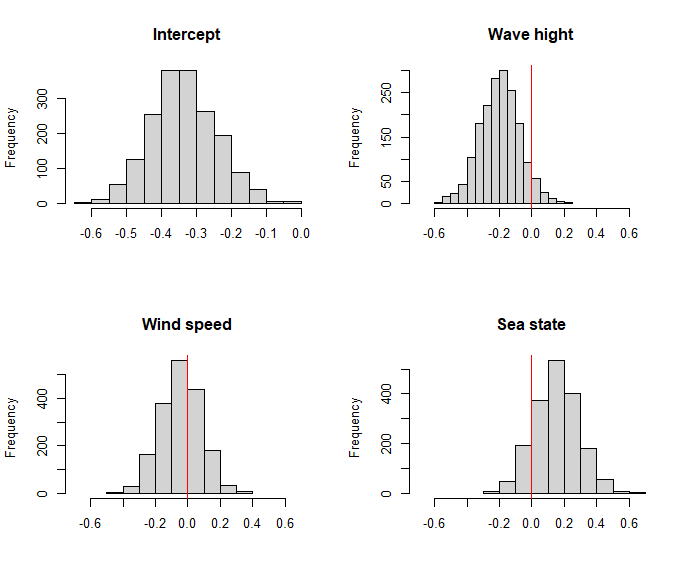


**Figure S4.** Posterior distributions for parameters controlling the effect of observational covariates on detection function for dusky dolphin. Red vertical lines denote zero.


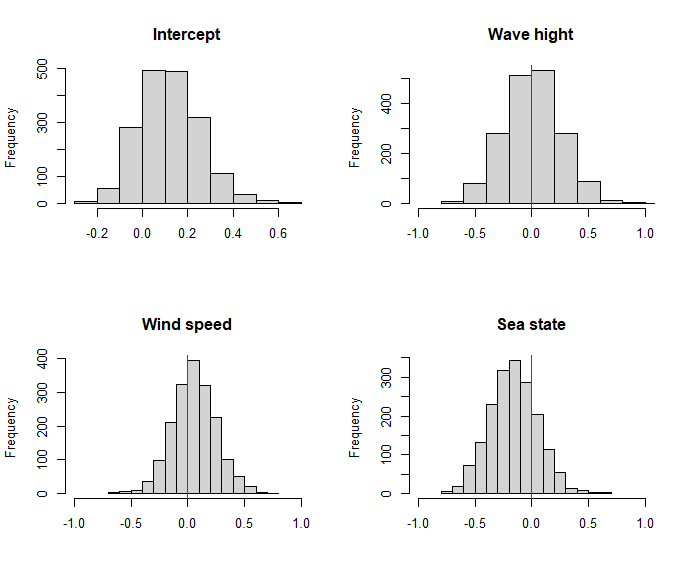


**Figure S5.** Posterior distributions for parameters controlling the effect of observational covariates on detection function for common dolphin. Red vertical lines denote zero.

**Table S2.** Results for model 1 fitted to fin whale data. Parameters for predictors are indicated by specific predictors’ name including quadratic terms. Parameters in bold indicate zero was not included in their posterior distribution. Psi (ψ) is the probability of a non-zero and prec corresponds to the precision (1/variance) of the normally distributed observation-level random effect.

| **Fin Whale - Model 1** | | | | | | | |
| --- | --- | --- | --- | --- | --- | --- | --- |
|  | **mean** | **sd** | **2.50%** | **25%** | **50%** | **75%** | **97.50%** |
| Intercept | -4.49013 | 0.391454 | -5.34853 | -4.70943 | -4.43936 | -4.21614 | -3.85012 |
| **SST** | **0.200159** | **0.085563** | **0.033511** | **0.142144** | **0.200506** | **0.256259** | **0.361769** |
| SST^2^ | -0.00807 | 0.072664 | -0.15254 | -0.05393 | -0.00759 | 0.041642 | 0.134727 |
| **LOGCHL** | **0.532293** | **0.099809** | **0.346135** | **0.463704** | **0.534292** | **0.599884** | **0.726111** |
| **LOGCHL^2^** | **-0.34907** | **0.083987** | **-0.52224** | **-0.40234** | **-0.34645** | **-0.29318** | **-0.18539** |
| **TG** | **-0.33086** | **0.128139** | **-0.57826** | **-0.41834** | **-0.32884** | **-0.24564** | **-0.0798** |
| TG^2^ | 0.029943 | 0.052709 | -0.07589 | -0.00433 | 0.030711 | 0.065567 | 0.131458 |
| SLO | 0.08145 | 0.103661 | -0.12147 | 0.013842 | 0.079318 | 0.151972 | 0.284013 |
| SLO^2^ | 0.037895 | 0.05964 | -0.07297 | -0.00555 | 0.036293 | 0.078979 | 0.156875 |
| **SSTA** | **-0.19749** | **0.085192** | **-0.36513** | **-0.25403** | **-0.19532** | **-0.14035** | **-0.03471** |
| SSTA^2^ | -0.02913 | 0.05508 | -0.13696 | -0.06504 | -0.02763 | 0.007135 | 0.075363 |
| Mean group size | 2.000323 | 0.078813 | 1.846808 | 1.944769 | 1.998668 | 2.05434 | 2.158228 |
| Variance group size | 2.190282 | 0.140848 | 1.953825 | 2.089196 | 2.175568 | 2.27546 | 2.488871 |
| psi | 0.262595 | 0.06684 | 0.176193 | 0.21786 | 0.247561 | 0.292602 | 0.433559 |
| prec | 1.756711 | 0.854459 | 0.637107 | 1.108382 | 1.612135 | 2.148235 | 3.988895 |
| A0 | 0.245721 | 0.075024 | 0.101557 | 0.196959 | 0.245984 | 0.293821 | 0.397129 |
| **Wave height** | **-0.24193** | **0.069433** | **-0.37599** | **-0.28824** | **-0.24149** | **-0.19702** | **-0.10931** |
| Wind speed | 0.050461 | 0.06814 | -0.08526 | 0.006305 | 0.05168 | 0.093817 | 0.185826 |
| Sea State | -0.08969 | 0.073489 | -0.23974 | -0.13764 | -0.08722 | -0.04036 | 0.054009 |
| bpv | 0.615 | 0.486731 | 0 | 0 | 1 | 1 | 1 |
| c.hat | 1.034799 | 0.104685 | 0.846513 | 0.971352 | 1.024194 | 1.099278 | 1.247219 |
| observed range | 6 | 0 | 6 | 6 | 6 | 6 | 6 |
| expected range | 5.942222 | 1.355201 | 4 | 5 | 6 | 7 | 9 |


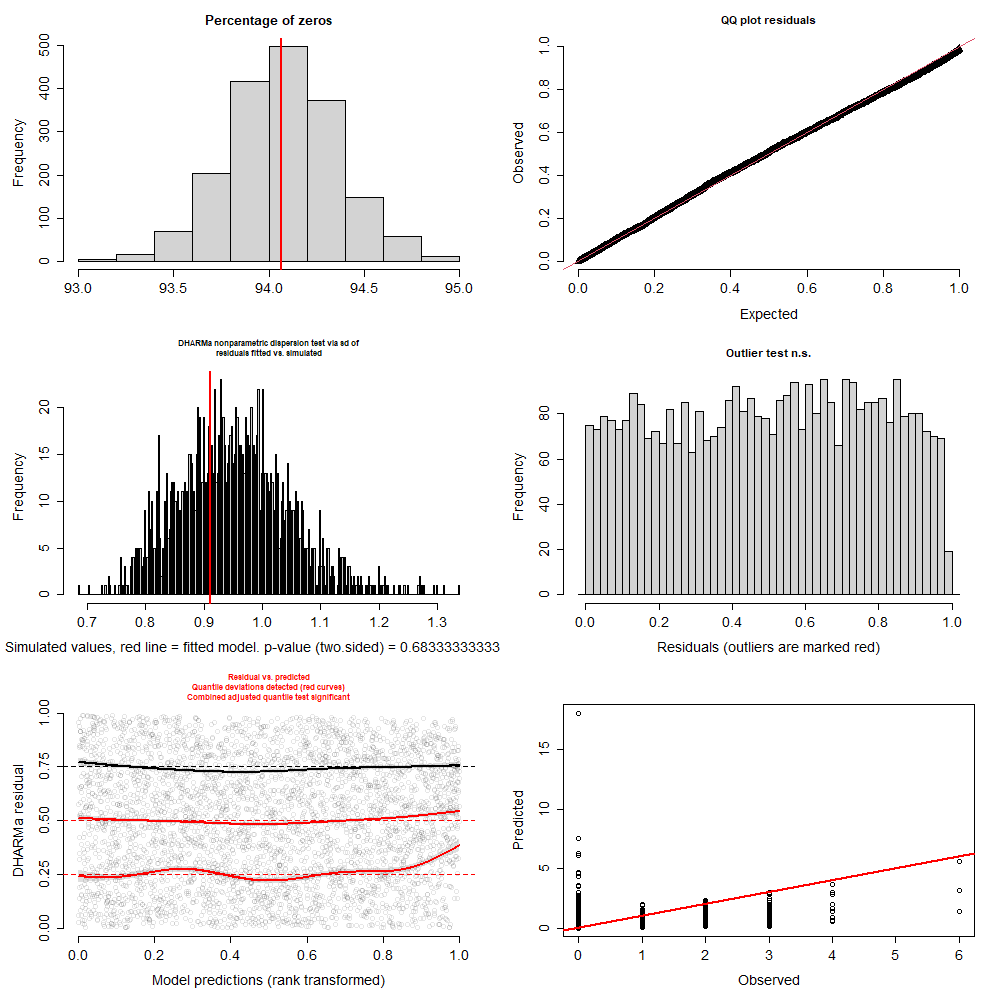


**Figure S6.** Fin whale – Model 1. Results for simulation-based and scaled residuals diagnostics for model-fit assessment. Left top panel shows the percentage of zeros in observed data (red line) and simulated data (histogram of posterior distribution). Right top panel show expected versus observed residual plot using scaled residuals. Left mid-panel show residual dispersion for observed data (red line) and simulated data (histogram of posterior distribution). Right mid-panel show result for outlier test, indicating now outliers detected. Left bottom panel show quantile deviation test, red lines indicate deviations detected. Right bottom panel show observed versus predicted plot.

**Table S3.** Results for model 2 fitted to fin whale data. Parameters for predictors are indicated by specific predictors’ name including quadratic terms. Parameters in bold indicate zero was not included in their posterior distribution. Psi (ψ) is the probability of a non-zero and prec corresponds to the precision (1/variance) of the normally distributed observation-level random effect.

| **Fin Whale - Model 2** | | | | | | | |
| --- | --- | --- | --- | --- | --- | --- | --- |
|  | **mean** | **sd** | **2.50%** | **25%** | **50%** | **75%** | **97.50%** |
| Intercept | -4.4121 | 0.503675 | -5.76642 | -4.63158 | -4.3148 | -4.07577 | -3.71394 |
| SSTLT | -0.07833 | 0.094193 | -0.26315 | -0.14268 | -0.07879 | -0.01835 | 0.111082 |
| **SSTLT^2^** | **-0.22153** | **0.086268** | **-0.39098** | **-0.27835** | **-0.22195** | **-0.16102** | **-0.05111** |
| **LOGCHL** | **0.52097** | **0.10318** | **0.321784** | **0.453005** | **0.52379** | **0.587877** | **0.730561** |
| **LOGCHL^2^** | **-0.35755** | **0.083558** | **-0.52293** | **-0.41427** | **-0.35824** | **-0.30112** | **-0.19503** |
| **TG** | **-0.28376** | **0.1303** | **-0.5419** | **-0.36952** | **-0.28531** | **-0.19358** | **-0.03495** |
| TG^2^ | 0.025499 | 0.052904 | -0.08093 | -0.0107 | 0.026735 | 0.060785 | 0.126882 |
| SLO | 0.050682 | 0.103398 | -0.14654 | -0.02031 | 0.053695 | 0.120096 | 0.253618 |
| SLO^2^ | 0.03926 | 0.059909 | -0.07055 | -0.00181 | 0.035616 | 0.079408 | 0.154047 |
| SSTA | -0.11607 | 0.080777 | -0.27129 | -0.17141 | -0.11577 | -0.06213 | 0.043393 |
| SSTA^2^ | -0.01979 | 0.056024 | -0.12825 | -0.05609 | -0.01968 | 0.016663 | 0.088762 |
| Mean group size | 2.002077 | 0.078641 | 1.850202 | 1.948236 | 2.004017 | 2.053573 | 2.157022 |
| Variance group size | 2.194365 | 0.13886 | 1.964714 | 2.094949 | 2.180042 | 2.279315 | 2.503051 |
| psi | 0.286092 | 0.100238 | 0.178969 | 0.22515 | 0.25854 | 0.309693 | 0.59405 |
| prec | 1.512032 | 0.739827 | 0.450714 | 0.985362 | 1.383977 | 1.927046 | 3.327136 |
| A0 | 0.25101 | 0.072427 | 0.119113 | 0.200795 | 0.250589 | 0.298786 | 0.391812 |
| **Wave height** | **-0.25185** | **0.071249** | **-0.38755** | **-0.3012** | **-0.25195** | **-0.20391** | **-0.10917** |
| Wind speed | 0.050301 | 0.069035 | -0.082 | 0.005172 | 0.048253 | 0.095544 | 0.192552 |
| Sea State | -0.08892 | 0.070429 | -0.22986 | -0.13551 | -0.08814 | -0.04261 | 0.050857 |
| bpv | 0.607222 | 0.488504 | 0 | 0 | 1 | 1 | 1 |
| c.hat | 1.034759 | 0.115157 | 0.83033 | 0.964657 | 1.023507 | 1.097252 | 1.289203 |
| observed range | 6 | 0 | 6 | 6 | 6 | 6 | 6 |
| expected range | 5.881111 | 1.32378 | 4 | 5 | 6 | 7 | 9 |

**
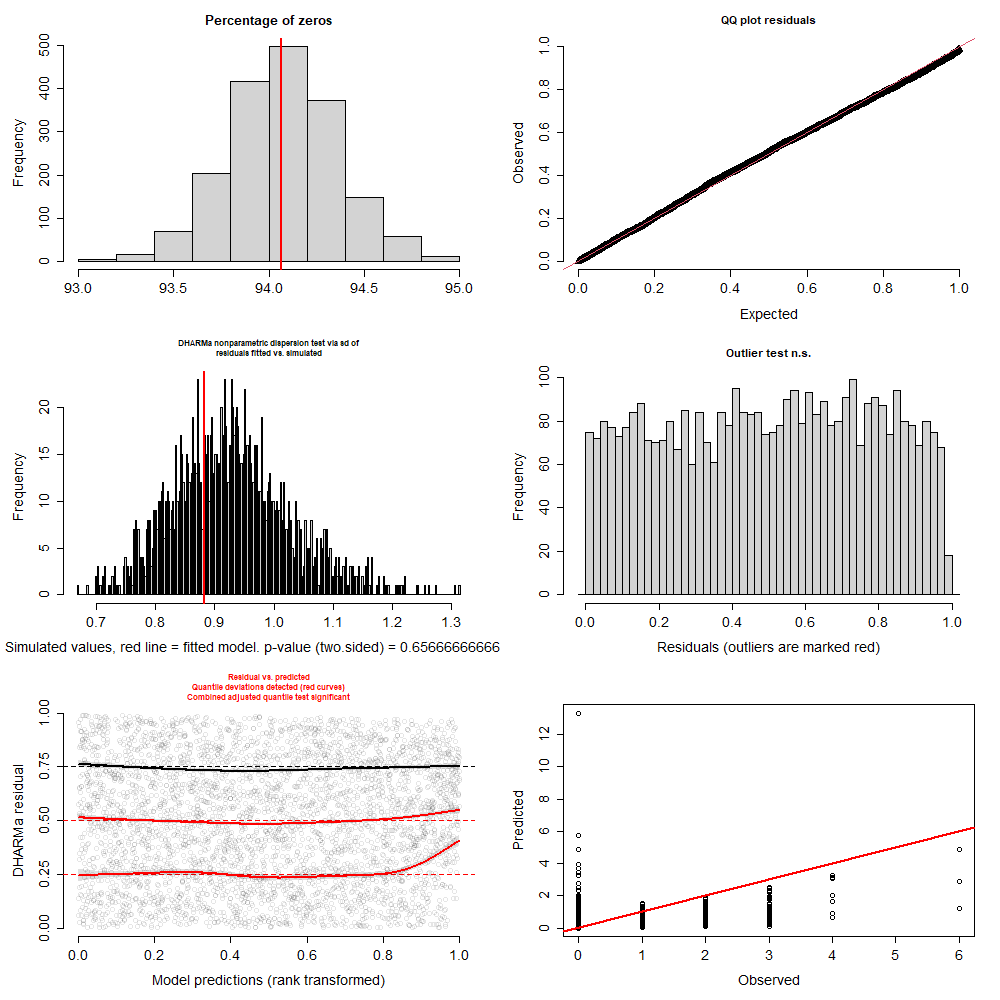
**

**Figure S7.** Fin whale – Model 2. Results for simulation-based and scaled residuals diagnostics for model-fit assessment. Left top panel shows the percentage of zeros in observed data (red line) and simulated data (histogram of posterior distribution). Right top panel show expected versus observed residual plot using scaled residuals. Left mid-panel show residual dispersion for observed data (red line) and simulated data (histogram of posterior distribution). Right mid-panel show result for outlier test, indicating now outliers detected. Left bottom panel show quantile deviation test, red lines indicate deviations detected. Right bottom panel show observed versus predicted plot.

**Table S4.** Results for model 3 fitted to fin whale data. Parameters for predictors are indicated by specific predictors’ name including quadratic terms. Parameters in bold indicate zero was not included in their posterior distribution. Psi (ψ) is the probability of a non-zero and prec corresponds to the precision (1/variance) of the normally distributed observation-level random effect.

| **Fin Whale - Model 3** | | | | | | | |
| --- | --- | --- | --- | --- | --- | --- | --- |
|  | **mean** | **sd** | **2.50%** | **25%** | **50%** | **75%** | **97.50%** |
| Intercept | -4.57397 | 0.416726 | -5.55886 | -4.81647 | -4.52946 | -4.27448 | -3.89324 |
| **SST** | **0.206955** | **0.087464** | **0.040568** | **0.146311** | **0.204006** | **0.264766** | **0.382187** |
| SST^2^ | -0.03675 | 0.072759 | -0.17401 | -0.08696 | -0.0378 | 0.013241 | 0.111562 |
| **CHLLT** | **0.632969** | **0.103619** | **0.425864** | **0.563589** | **0.631464** | **0.703829** | **0.830891** |
| **CHLLT^2^** | **-0.3427** | **0.089268** | **-0.52008** | **-0.40088** | **-0.34273** | **-0.28122** | **-0.16998** |
| **TG** | **-0.34629** | **0.127951** | **-0.59183** | **-0.43711** | **-0.34399** | **-0.2587** | **-0.09615** |
| TG^2^ | 0.034014 | 0.050944 | -0.07035 | 0.000579 | 0.035323 | 0.068442 | 0.13401 |
| SLO | 0.109652 | 0.104915 | -0.09197 | 0.037556 | 0.110441 | 0.182547 | 0.311789 |
| SLO^2^ | 0.028748 | 0.058345 | -0.08181 | -0.01224 | 0.025618 | 0.069128 | 0.142602 |
| **SSTA** | **-0.17454** | **0.084049** | **-0.3435** | **-0.22844** | **-0.17197** | **-0.11605** | **-0.01506** |
| SSTA^2^ | -0.01665 | 0.053134 | -0.12373 | -0.05168 | -0.01701 | 0.019938 | 0.086746 |
| Mean group size | 2.005659 | 0.076193 | 1.863406 | 1.953259 | 2.006213 | 2.055012 | 2.161108 |
| Variance group size | 2.198442 | 0.138464 | 1.958511 | 2.096701 | 2.186663 | 2.283073 | 2.502038 |
| psi | 0.281427 | 0.072316 | 0.185772 | 0.229767 | 0.265678 | 0.316348 | 0.464302 |
| prec | 1.735992 | 0.868861 | 0.638204 | 1.114325 | 1.497358 | 2.177124 | 3.994453 |
| A0 | 0.239359 | 0.072011 | 0.103383 | 0.189915 | 0.237807 | 0.28897 | 0.383758 |
| **Wave height** | **-0.23787** | **0.070226** | **-0.37629** | **-0.28477** | **-0.23813** | **-0.18811** | **-0.10744** |
| Wind speed | 0.061765 | 0.068547 | -0.06857 | 0.014382 | 0.060375 | 0.106797 | 0.197129 |
| Sea State | -0.08914 | 0.074186 | -0.22935 | -0.13864 | -0.08811 | -0.03885 | 0.051859 |
| bpv | 0.658333 | 0.4744 | 0 | 0 | 1 | 1 | 1 |
| c.hat | 1.044937 | 0.114346 | 0.838939 | 0.974183 | 1.033124 | 1.108995 | 1.29578 |
| observed range | 6 | 0 | 6 | 6 | 6 | 6 | 6 |
| expected range | 5.919444 | 1.28819 | 4 | 5 | 6 | 7 | 9 |

**
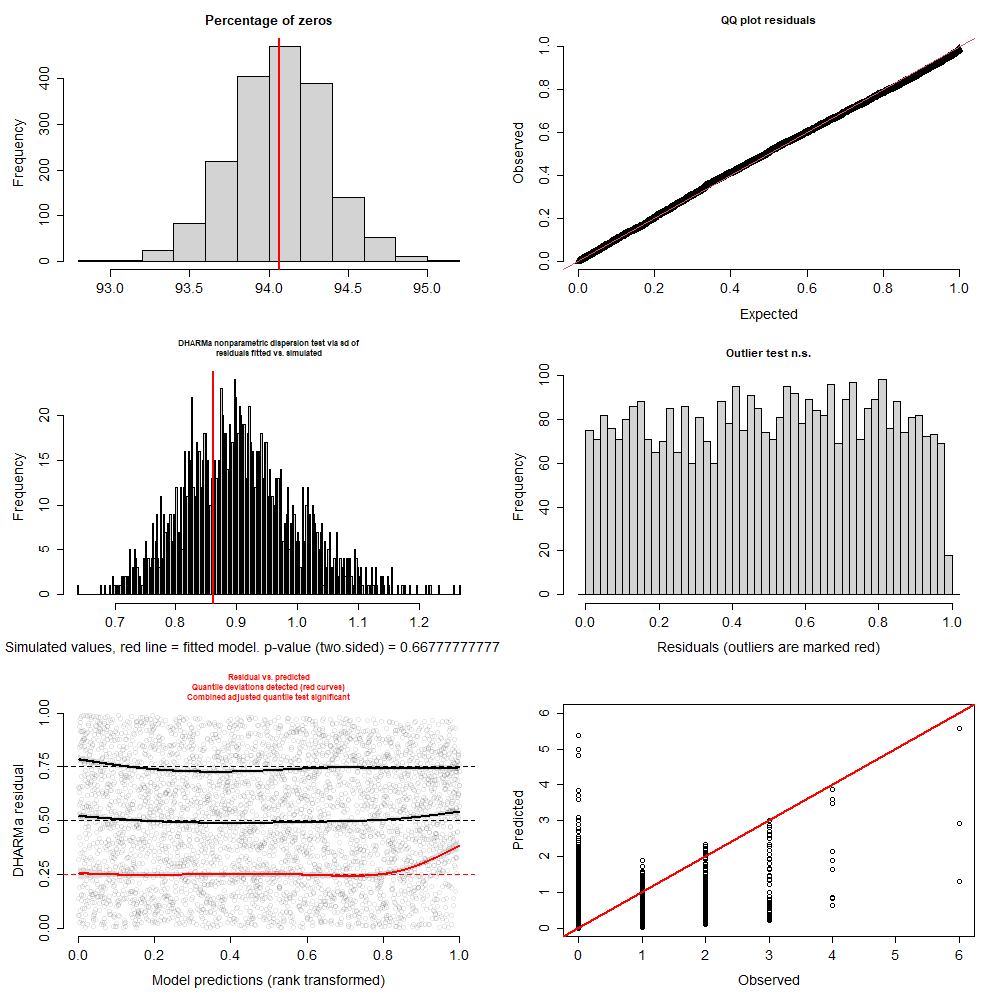
**

**Figure S8.** Fin whale – Model 3. Results for simulation-based and scaled residuals diagnostics for model-fit assessment. Left top panel shows the percentage of zeros in observed data (red line) and simulated data (histogram of posterior distribution). Right top panel show expected versus observed residual plot using scaled residuals. Left mid-panel show residual dispersion for observed data (red line) and simulated data (histogram of posterior distribution). Right mid-panel show result for outlier test, indicating now outliers detected. Left bottom panel show quantile deviation test, red lines indicate deviations detected. Right bottom panel show observed versus predicted plot.

**Table S5.** Results for model 4 fitted to fin whale data. Parameters for predictors are indicated by specific predictors’ name including quadratic terms. Parameters in bold indicate zero was not included in their posterior distribution. Psi (ψ) is the probability of a non-zero and prec corresponds to the precision (1/variance) of the normally distributed observation-level random effect.

| **Fin Whale - Model 4** | | | | | | | |
| --- | --- | --- | --- | --- | --- | --- | --- |
|  | **mean** | **sd** | **2.50%** | **25%** | **50%** | **75%** | **97.50%** |
| Intercept | -4.47658 | 0.479234 | -5.62842 | -4.72514 | -4.39995 | -4.14596 | -3.76501 |
| SSTLT | -0.01713 | 0.095979 | -0.19915 | -0.08257 | -0.01587 | 0.046732 | 0.165747 |
| **SSTLT^2^** | **-0.21057** | **0.086568** | **-0.38403** | **-0.26643** | **-0.20873** | **-0.15229** | **-0.04125** |
| **CHLLT** | **0.621448** | **0.106677** | **0.423711** | **0.548167** | **0.619809** | **0.691005** | **0.834325** |
| **CHLLT^2^** | **-0.35726** | **0.08891** | **-0.53831** | **-0.41411** | **-0.35721** | **-0.29844** | **-0.18764** |
| **TG** | **-0.29881** | **0.130599** | **-0.55762** | **-0.38462** | **-0.29795** | **-0.21118** | **-0.05407** |
| TG^2^ | 0.027797 | 0.051981 | -0.07776 | -0.00502 | 0.028528 | 0.062434 | 0.130139 |
| SLO | 0.076102 | 0.108409 | -0.14029 | 0.001812 | 0.076778 | 0.147542 | 0.29096 |
| SLO^2^ | 0.037327 | 0.058845 | -0.06984 | -0.00482 | 0.033321 | 0.078931 | 0.155293 |
| SSTA | -0.0989 | 0.080429 | -0.25593 | -0.15385 | -0.09953 | -0.04379 | 0.057579 |
| SSTA^2^ | -0.01373 | 0.054587 | -0.12177 | -0.0502 | -0.01377 | 0.023418 | 0.09414 |
| Mean group size | 2.002722 | 0.078067 | 1.855932 | 1.950177 | 2.001534 | 2.055358 | 2.155704 |
| Variance group size | 2.194459 | 0.142722 | 1.950222 | 2.093494 | 2.179616 | 2.27812 | 2.51973 |
| psi | 0.295327 | 0.096167 | 0.18535 | 0.233003 | 0.27126 | 0.328723 | 0.537331 |
| prec | 1.572296 | 0.842869 | 0.512628 | 0.944965 | 1.334069 | 1.99033 | 3.703727 |
| A0 | 0.241719 | 0.071427 | 0.108464 | 0.190531 | 0.240814 | 0.290925 | 0.381768 |
| **Wave height** | **-0.24875** | **0.07095** | **-0.38156** | **-0.29932** | **-0.24872** | **-0.20111** | **-0.10988** |
| Wind speed | 0.06068 | 0.068483 | -0.06701 | 0.013597 | 0.059369 | 0.107159 | 0.197073 |
| Sea State | -0.08709 | 0.074213 | -0.22906 | -0.13807 | -0.08885 | -0.03469 | 0.054828 |
| bpv | 0.645556 | 0.478478 | 0 | 0 | 1 | 1 | 1 |
| c.hat | 1.047529 | 0.11927 | 0.850376 | 0.97278 | 1.037409 | 1.113339 | 1.303761 |
| observed range | 6 | 0 | 6 | 6 | 6 | 6 | 6 |
| expected range | 5.934444 | 1.288394 | 4 | 5 | 6 | 7 | 9 |

**
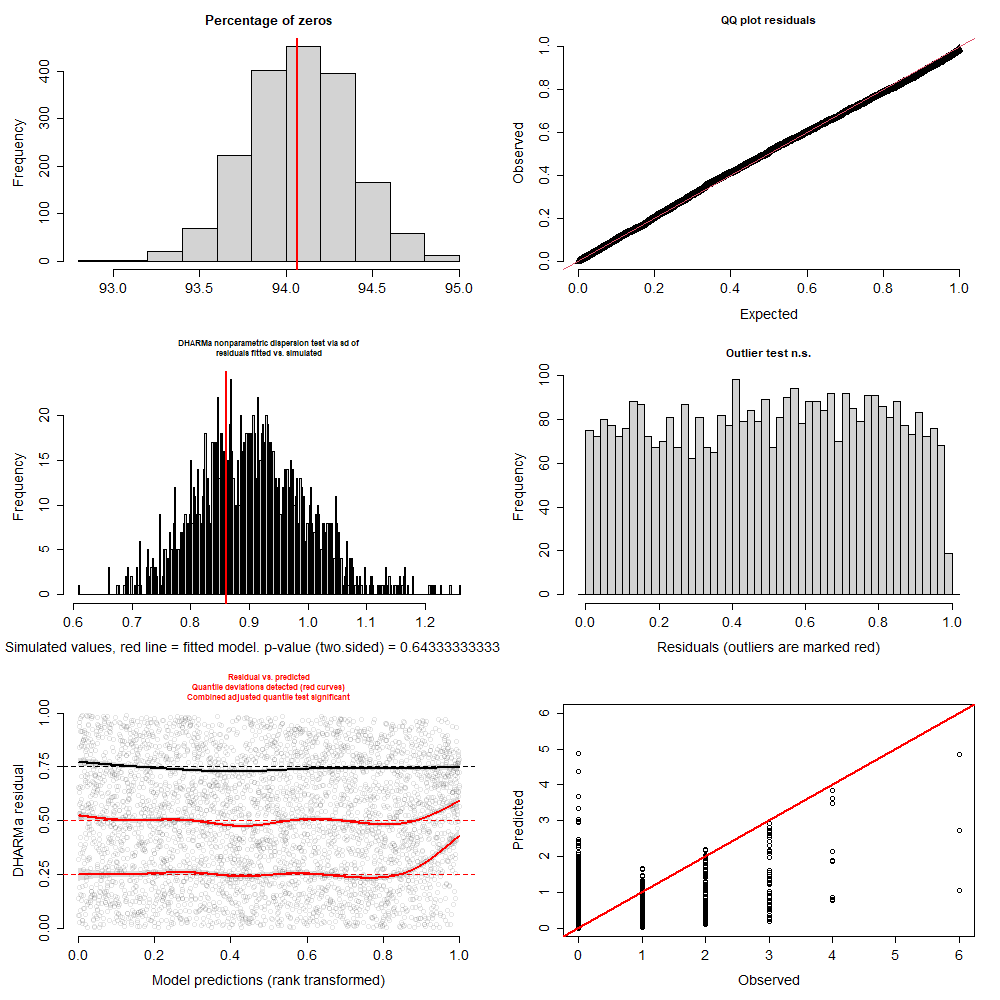
**

**Figure S9.** Fin whale – Model 4. Results for simulation-based and scaled residuals diagnostics for model-fit assessment. Left top panel shows the percentage of zeros in observed data (red line) and simulated data (histogram of posterior distribution). Right top panel show expected versus observed residual plot using scaled residuals. Left mid-panel show residual dispersion for observed data (red line) and simulated data (histogram of posterior distribution). Right mid-panel show result for outlier test, indicating now outliers detected. Left bottom panel show quantile deviation test, red lines indicate deviations detected. Right bottom panel show observed versus predicted plot.

**Table S6.** Results for model 1 fitted to blue whale data. Parameters for predictors are indicated by specific predictors’ name including quadratic terms. Parameters in bold indicate zero was not included in their posterior distribution. Psi (ψ) is the probability of a non-zero and prec corresponds to the precision (1/variance) of the normally distributed observation-level random effect.

| **Blue whale - Model 1** | | | | | | | |
| --- | --- | --- | --- | --- | --- | --- | --- |
|  | **mean** | **sd** | **2.50%** | **25%** | **50%** | **75%** | **97.50%** |
| Intercept | -7.44547 | 1.253039 | -9.57318 | -8.4707 | -7.48224 | -6.54699 | -4.99676 |
| CHLLT | -0.44109 | 0.231554 | -0.93495 | -0.59053 | -0.43522 | -0.28078 | 0.006317 |
| **CHLLT^2^** | **-0.51957** | **0.251881** | **-1.04962** | **-0.67297** | **-0.50641** | **-0.34937** | **-0.05792** |
| TG | 0.134679 | 0.287823 | -0.42384 | -0.0664 | 0.141619 | 0.325733 | 0.694442 |
| TG^2^ | -0.09385 | 0.123074 | -0.34991 | -0.16901 | -0.0878 | -0.00891 | 0.129384 |
| **JD** | **0.711556** | **0.18534** | **0.356886** | **0.589609** | **0.704796** | **0.83182** | **1.085552** |
| JD^2^ | 0.044382 | 0.296602 | -0.54054 | -0.15603 | 0.042507 | 0.241172 | 0.629786 |
| Mean group size | 1.695182 | 0.163799 | 1.390239 | 1.583138 | 1.688583 | 1.796453 | 2.037731 |
| Variance group size | 1.757592 | 0.186849 | 1.424971 | 1.633505 | 1.744531 | 1.870365 | 2.166536 |
| psi | 0.416494 | 0.243963 | 0.091161 | 0.212223 | 0.367266 | 0.596373 | 0.950332 |
| prec | 0.648967 | 0.706122 | 0.229647 | 0.293282 | 0.397733 | 0.628563 | 3.262475 |
| A0 | 0.333184 | 0.153227 | 0.064337 | 0.226451 | 0.323122 | 0.431209 | 0.65711 |
| **Wave height** | **-0.437** | **0.185642** | **-0.7979** | **-0.55997** | **-0.437** | **-0.31174** | **-0.06469** |
| Wind speed | 0.130814 | 0.173494 | -0.21518 | 0.01548 | 0.131645 | 0.249184 | 0.47259 |
| Sea State | -0.094 | 0.182154 | -0.45566 | -0.21775 | -0.08953 | 0.030488 | 0.246558 |
| bpv | 0.595556 | 0.490921 | 0 | 0 | 1 | 1 | 1 |
| c.hat | 1.264295 | 0.951214 | 0.400326 | 0.861215 | 1.078168 | 1.390132 | 3.263369 |
| observed range | 3 | 0 | 3 | 3 | 3 | 3 | 3 |
| expected range | 3.149444 | 1.011001 | 2 | 2 | 3 | 4 | 6 |

**
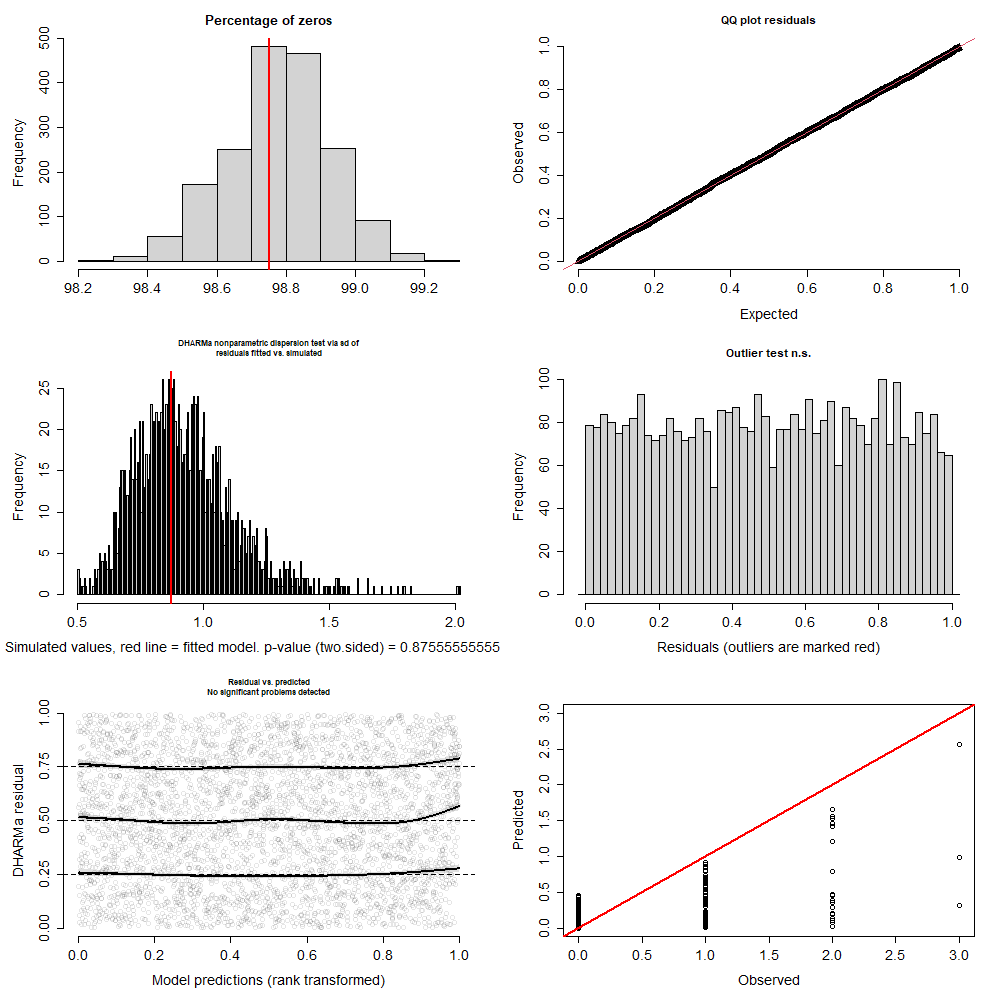
**

**Figure S10.** Blue whale – Model 1. Results for simulation-based and scaled residuals diagnostics for model-fit assessment. Left top panel shows the percentage of zeros in observed data (red line) and simulated data (histogram of posterior distribution). Right top panel show expected versus observed residual plot using scaled residuals. Left mid-panel show residual dispersion for observed data (red line) and simulated data (histogram of posterior distribution). Right mid-panel show result for outlier test, indicating now outliers detected. Left bottom panel show quantile deviation test, red lines indicate deviations detected. Right bottom panel show observed versus predicted plot.

**Table S7.** Results for model 2 fitted to blue whale data. Parameters for predictors are indicated by specific predictors’ name including quadratic terms. Parameters in bold indicate zero was not included in their posterior distribution. Psi (ψ) is the probability of a non-zero and prec corresponds to the precision (1/variance) of the normally distributed observation-level random effect.

| **Blue whale - Model 2** | | | | | | | |
| --- | --- | --- | --- | --- | --- | --- | --- |
|  | **mean** | **sd** | **2.50%** | **25%** | **50%** | **75%** | **97.50%** |
| Intercept | -7.61364 | 1.181652 | -9.47168 | -8.49435 | -7.81956 | -6.89062 | -4.99875 |
| LOGCHL | -0.43015 | 0.238911 | -0.88535 | -0.58614 | -0.42571 | -0.2696 | 0.032533 |
| **LOGCHL^2^** | **-0.65225** | **0.253189** | **-1.17135** | **-0.81028** | **-0.64165** | **-0.47939** | **-0.19183** |
| TG | 0.083159 | 0.280192 | -0.47187 | -0.09887 | 0.078968 | 0.268722 | 0.6415 |
| TG^2^ | -0.06699 | 0.121445 | -0.33591 | -0.14057 | -0.06096 | 0.01501 | 0.153417 |
| **JD** | 0.701621 | 0.190703 | 0.348491 | 0.567739 | 0.697507 | 0.829673 | 1.08684 |
| JD^2^ | 0.115131 | 0.286768 | -0.45757 | -0.07194 | 0.113514 | 0.300404 | 0.689417 |
| Mean group size | 1.689344 | 0.169674 | 1.38526 | 1.572687 | 1.67989 | 1.799373 | 2.046788 |
| Variance group size | 1.753556 | 0.193052 | 1.419948 | 1.618582 | 1.741219 | 1.874705 | 2.170329 |
| psi | 0.442045 | 0.22995 | 0.087759 | 0.251738 | 0.424672 | 0.608869 | 0.914136 |
| prec | 0.647492 | 0.765205 | 0.229367 | 0.286927 | 0.366638 | 0.549474 | 3.355442 |
| A0 | 0.331562 | 0.155616 | 0.056263 | 0.224006 | 0.320031 | 0.433581 | 0.651862 |
| **Wave height** | **-0.41193** | **0.184147** | **-0.76447** | **-0.53967** | **-0.41584** | **-0.29143** | **-0.04048** |
| Wind speed | 0.131931 | 0.17256 | -0.21505 | 0.014952 | 0.137086 | 0.24816 | 0.478435 |
| Sea State | -0.1045 | 0.186604 | -0.47861 | -0.2269 | -0.10426 | 0.023574 | 0.267807 |
| bpv | 0.593889 | 0.491242 | 0 | 0 | 1 | 1 | 1 |
| c.hat | 1.323436 | 1.801144 | 0.396953 | 0.85896 | 1.088791 | 1.43699 | 3.250644 |
| observed range | 3 | 0 | 3 | 3 | 3 | 3 | 3 |
| expected range | 3.142778 | 1.041738 | 2 | 2 | 3 | 4 | 6 |

**
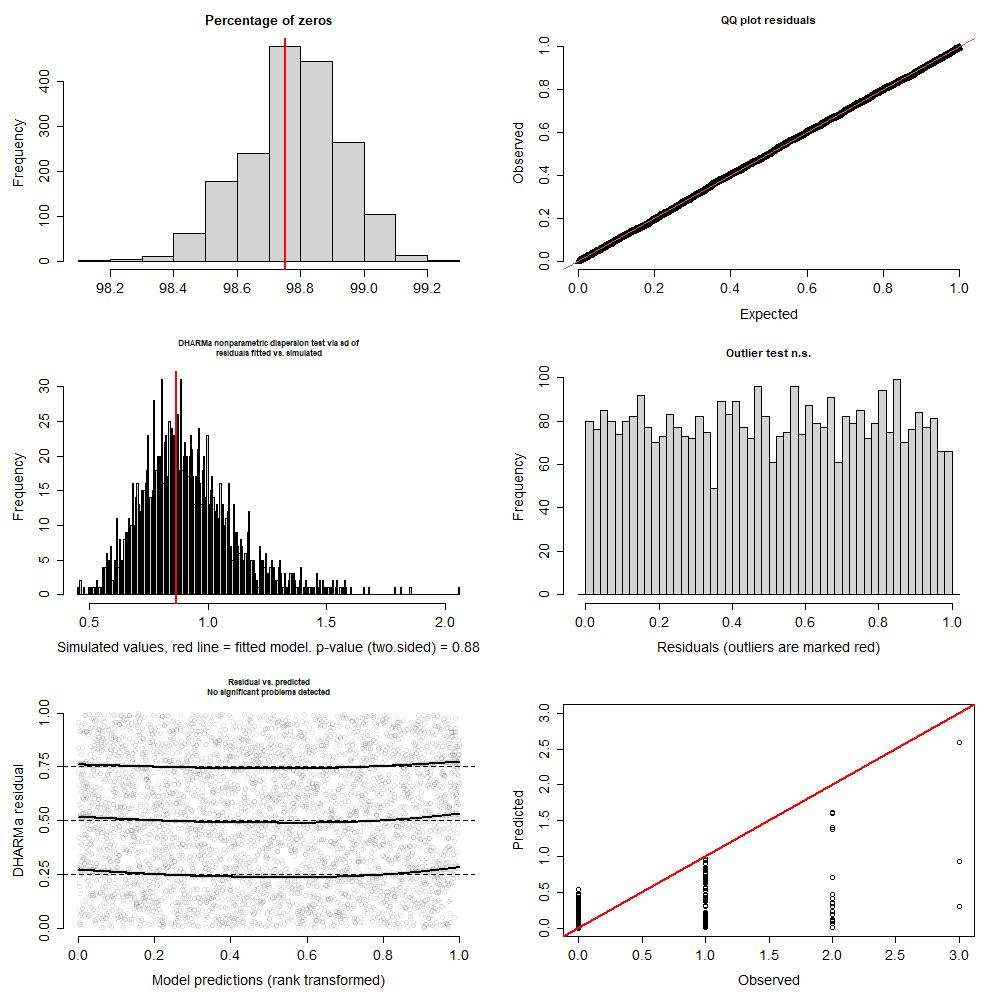
**

**Figure S11.** Blue whale – Model 2. Results for simulation-based and scaled residuals diagnostics for model-fit assessment. Left top panel shows the percentage of zeros in observed data (red line) and simulated data (histogram of posterior distribution). Right top panel show expected versus observed residual plot using scaled residuals. Left mid-panel show residual dispersion for observed data (red line) and simulated data (histogram of posterior distribution). Right mid-panel show result for outlier test, indicating now outliers detected. Left bottom panel show quantile deviation test, red lines indicate deviations detected. Right bottom panel show observed versus predicted plot.

**Table S8.** Results for model 1 fitted to sperm whale data. Parameters for predictors are indicated by specific predictors’ name including quadratic terms. Parameters in bold indicate zero was not included in their posterior distribution. Psi (ψ) is the probability of a non-zero and prec corresponds to the precision (1/variance) of the normally distributed observation-level random effect.

| **Sperm whale - Model 1** | | | | | | | |
| --- | --- | --- | --- | --- | --- | --- | --- |
|  | **mean** | **sd** | **2.50%** | **25%** | **50%** | **75%** | **97.50%** |
| Intercept | -5.38531 | 0.918617 | -7.41284 | -5.96842 | -5.26909 | -4.69748 | -3.87157 |
| SSTLT | 0.02785 | 0.202446 | -0.37829 | -0.10889 | 0.033639 | 0.167457 | 0.426007 |
| SSTLT^2^ | -0.04083 | 0.188966 | -0.40126 | -0.17377 | -0.03715 | 0.085168 | 0.322689 |
| **DEPTH** | **-1.41877** | **0.290615** | **-2.01725** | **-1.61077** | **-1.40607** | **-1.22072** | **-0.88583** |
| **DEPTH^2^** | **-0.51172** | **0.17791** | **-0.86996** | **-0.62756** | **-0.51103** | **-0.39083** | **-0.16966** |
| SLO | 0.111319 | 0.207541 | -0.30356 | -0.03072 | 0.105658 | 0.248982 | 0.516983 |
| SLO^2^ | -0.1767 | 0.137774 | -0.4706 | -0.261 | -0.17049 | -0.07725 | 0.064846 |
| Mean group size | 2.689018 | 0.159451 | 2.388579 | 2.577478 | 2.686234 | 2.79591 | 3.002468 |
| Variance group size | 3.020484 | 0.324235 | 2.537371 | 2.794549 | 2.965721 | 3.188507 | 3.786102 |
| psi | 0.138852 | 0.071271 | 0.060048 | 0.088489 | 0.117866 | 0.16673 | 0.332589 |
| prec | 0.813358 | 0.573167 | 0.251467 | 0.437562 | 0.642812 | 0.981727 | 2.423023 |
| A0 | 0.333136 | 0.121473 | 0.115244 | 0.250561 | 0.327191 | 0.4079 | 0.585977 |
| **Wave height** | **-0.33224** | **0.123202** | **-0.56873** | **-0.41578** | **-0.33485** | **-0.24883** | **-0.09493** |
| Wind speed | 0.092478 | 0.117589 | -0.12937 | 0.014599 | 0.089048 | 0.171881 | 0.320326 |
| Sea State | -0.01633 | 0.128647 | -0.27798 | -0.10173 | -0.01431 | 0.075796 | 0.225188 |
| bpv | 0.506111 | 0.500102 | 0 | 0 | 1 | 1 | 1 |
| c.hat | 1.042765 | 0.333755 | 0.564766 | 0.875322 | 1.002936 | 1.165934 | 1.719342 |
| observed range | 7 | 0 | 7 | 7 | 7 | 7 | 7 |
| expected range | 6.85 | 1.655671 | 4 | 6 | 7 | 8 | 11 |

**
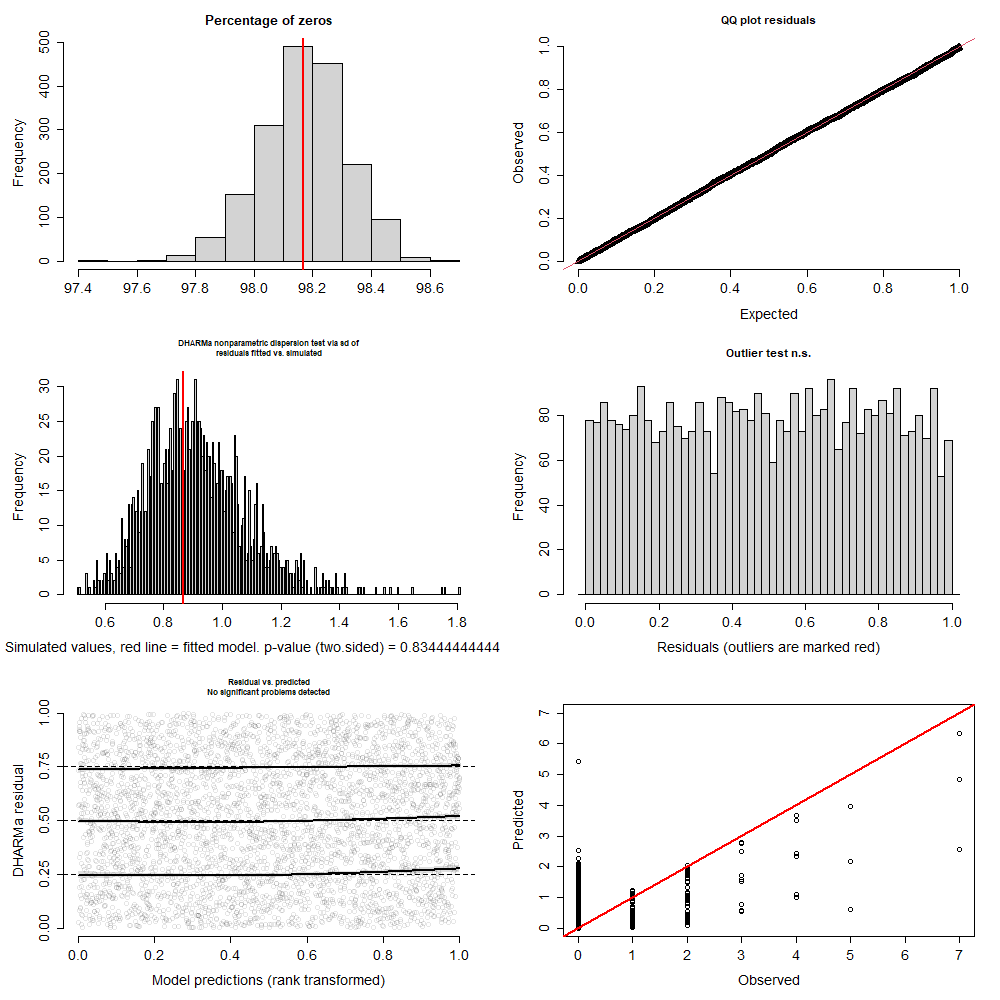
**

**Figure S12.** Sperm whale – Model 1. Results for simulation-based and scaled residuals diagnostics for model-fit assessment. Left top panel shows the percentage of zeros in observed data (red line) and simulated data (histogram of posterior distribution). Right top panel show expected versus observed residual plot using scaled residuals. Left mid-panel show residual dispersion for observed data (red line) and simulated data (histogram of posterior distribution). Right mid-panel show result for outlier test, indicating now outliers detected. Left bottom panel show quantile deviation test, red lines indicate deviations detected. Right bottom panel show observed versus predicted plot.

**Table S9.** Results for model 2 fitted to sperm whale data. Parameters for predictors are indicated by specific predictors’ name including quadratic terms. Parameters in bold indicate zero was not included in their posterior distribution. Psi (ψ) is the probability of a non-zero and prec corresponds to the precision (1/variance) of the normally distributed observation-level random effect.

| **Sperm whale - Model 2** | | | | | | | |
| --- | --- | --- | --- | --- | --- | --- | --- |
|  | **mean** | **sd** | **2.50%** | **25%** | **50%** | **75%** | **97.50%** |
| Intercept | -7.61146 | 0.735832 | -8.84144 | -8.1504 | -7.69744 | -7.14089 | -5.96968 |
| **SST** | **-1.24715** | **0.228244** | **-1.71061** | **-1.39668** | **-1.24282** | **-1.10052** | **-0.81228** |
| SST^2^ | -0.01616 | 0.19028 | -0.39693 | -0.13685 | -0.01139 | 0.111386 | 0.343302 |
| **DEPTH** | **-1.81176** | **0.306606** | **-2.46431** | **-2.0089** | **-1.79736** | **-1.60772** | **-1.24638** |
| **DEPTH^2^** | **-0.73692** | **0.193394** | **-1.14257** | **-0.86088** | **-0.72994** | **-0.6019** | **-0.38714** |
| SLO | 0.227473 | 0.209324 | -0.18293 | 0.090277 | 0.224684 | 0.371705 | 0.650881 |
| SLO^2^ | -0.19069 | 0.132313 | -0.47761 | -0.27346 | -0.18185 | -0.09285 | 0.032107 |
| Mean group size | 2.689819 | 0.156378 | 2.395915 | 2.581197 | 2.686528 | 2.796858 | 2.994346 |
| Variance group size | 3.013828 | 0.321964 | 2.521833 | 2.787668 | 2.968523 | 3.177573 | 3.818083 |
| psi | 0.420139 | 0.154978 | 0.176989 | 0.304953 | 0.402952 | 0.514743 | 0.770759 |
| prec | 0.30962 | 0.096942 | 0.225958 | 0.249018 | 0.281282 | 0.335626 | 0.551742 |
| A0 | 0.327844 | 0.120739 | 0.094635 | 0.247822 | 0.324984 | 0.412564 | 0.572666 |
| **Wave height** | **-0.32996** | **0.122101** | **-0.56257** | **-0.41209** | **-0.33264** | **-0.24604** | **-0.08544** |
| Wind speed | 0.108007 | 0.11889 | -0.11399 | 0.027175 | 0.0996 | 0.190395 | 0.349557 |
| Sea State | -0.02652 | 0.124602 | -0.26768 | -0.11059 | -0.02367 | 0.056731 | 0.211924 |
| bpv | 0.515556 | 0.499897 | 0 | 0 | 1 | 1 | 1 |
| c.hat | 1.213522 | 1.087451 | 0.343347 | 0.824589 | 1.013544 | 1.275516 | 3.487673 |
| observed range | 7 | 0 | 7 | 7 | 7 | 7 | 7 |
| expected range | 7.102778 | 1.681734 | 4 | 6 | 7 | 8 | 11 |

**
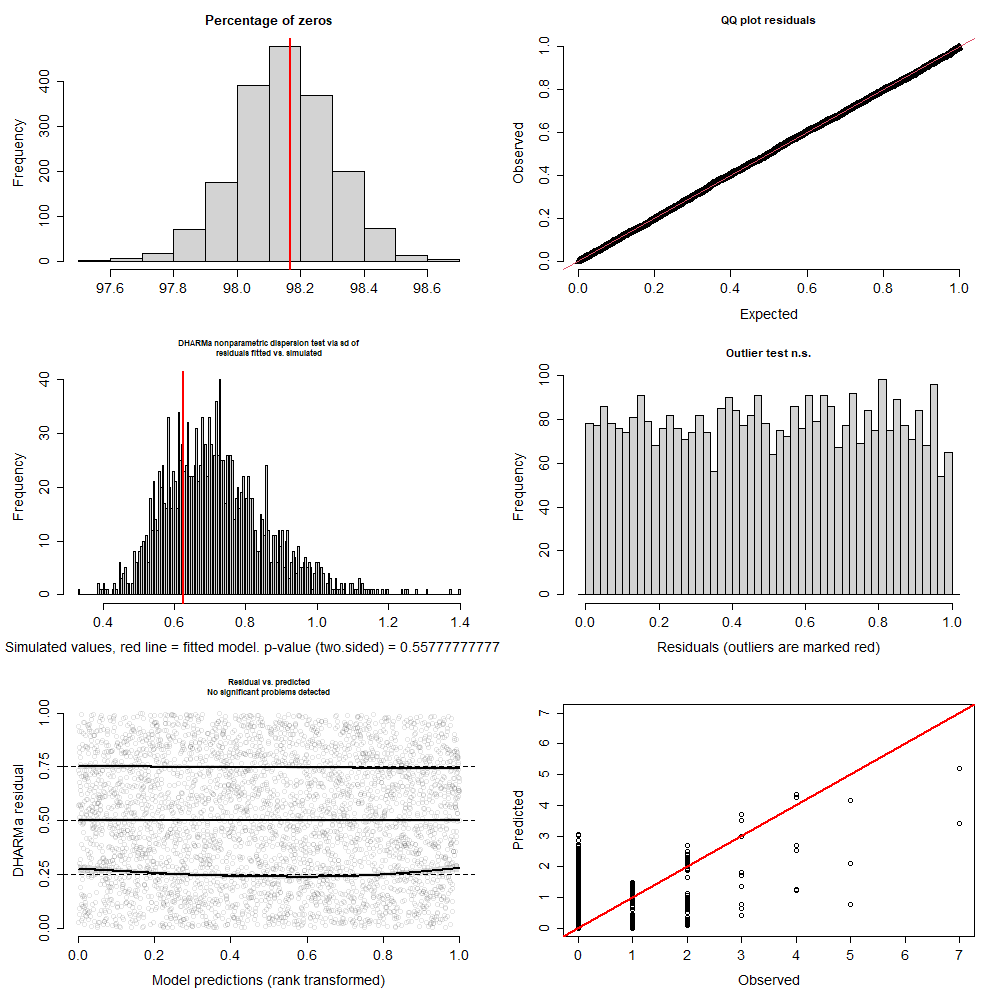
**

**Figure S13.** Sperm whale – Model 2. Results for simulation-based and scaled residuals diagnostics for model-fit assessment. Left top panel shows the percentage of zeros in observed data (red line) and simulated data (histogram of posterior distribution). Right top panel show expected versus observed residual plot using scaled residuals. Left mid-panel show residual dispersion for observed data (red line) and simulated data (histogram of posterior distribution). Right mid-panel show result for outlier test, indicating now outliers detected. Left bottom panel show quantile deviation test, red lines indicate deviations detected. Right bottom panel show observed versus predicted plot.

**Table S10.** Results for model 1 fitted to dusky dolphin data. Parameters for predictors are indicated by specific predictors’ name including quadratic terms. Parameters in bold indicate zero was not included in their posterior distribution. Psi (ψ) is the probability of a non-zero and prec corresponds to the precision (1/variance) of the normally distributed observation-level random effect.

| **Dusky dolphin - Model 1** | | | | | | | |
| --- | --- | --- | --- | --- | --- | --- | --- |
|  | **mean** | **sd** | **2.50%** | **25%** | **50%** | **75%** | **97.50%** |
| Intercept | -9.3206 | 1.186888 | -11.6752 | -10.1504 | -9.26449 | -8.508 | -7.11616 |
| **SSTLT** | **1.866241** | **0.356542** | **1.249588** | **1.620568** | **1.844293** | **2.087516** | **2.619399** |
| **SSTLT^2^** | **-0.96802** | **0.369436** | **-1.76198** | **-1.20489** | **-0.93974** | **-0.71286** | **-0.31365** |
| **DEPTH** | **4.373069** | **0.971437** | **2.876941** | **3.700482** | **4.253252** | **4.850211** | **6.712385** |
| DEPTH^2^ | 0.831254 | 0.76219 | -1.12749 | 0.427942 | 0.961203 | 1.347764 | 2.025603 |
| SLO | 0.056704 | 0.270914 | -0.45881 | -0.12512 | 0.06138 | 0.239536 | 0.589019 |
| SLO^2^ | -0.3087 | 0.228642 | -0.79448 | -0.45195 | -0.28897 | -0.14034 | 0.067577 |
| Mean group size | 18.61218 | 1.2774 | 16.33376 | 17.73167 | 18.53054 | 19.44915 | 21.19195 |
| Variance group size | 272.6842 | 46.68879 | 199.921 | 239.1607 | 268.1178 | 300.3799 | 377.3196 |
| psi | 0.388035 | 0.149617 | 0.191896 | 0.277427 | 0.355614 | 0.467341 | 0.785105 |
| prec | 0.502094 | 0.23967 | 0.236506 | 0.329166 | 0.428732 | 0.623013 | 1.121146 |
| A0 | -0.31943 | 0.089993 | -0.48972 | -0.38098 | -0.3244 | -0.26052 | -0.13116 |
| Wave height | -0.1493 | 0.110354 | -0.36766 | -0.22021 | -0.15069 | -0.07541 | 0.063671 |
| Wind speed | 0.135374 | 0.116688 | -0.098 | 0.057553 | 0.134673 | 0.215997 | 0.354454 |
| Sea State | -0.15665 | 0.110462 | -0.37523 | -0.2309 | -0.15728 | -0.08156 | 0.071505 |
| bpv | 0.573333 | 0.49473 | 0 | 0 | 1 | 1 | 1 |
| c.hat | 1.743525 | 3.296345 | 0.35161 | 0.888263 | 1.033963 | 1.394566 | 8.273868 |
| observed range | 6 | 0 | 6 | 6 | 6 | 6 | 6 |
| expected range | 7.668333 | 1.628504 | 5 | 7 | 7 | 9 | 12 |

**
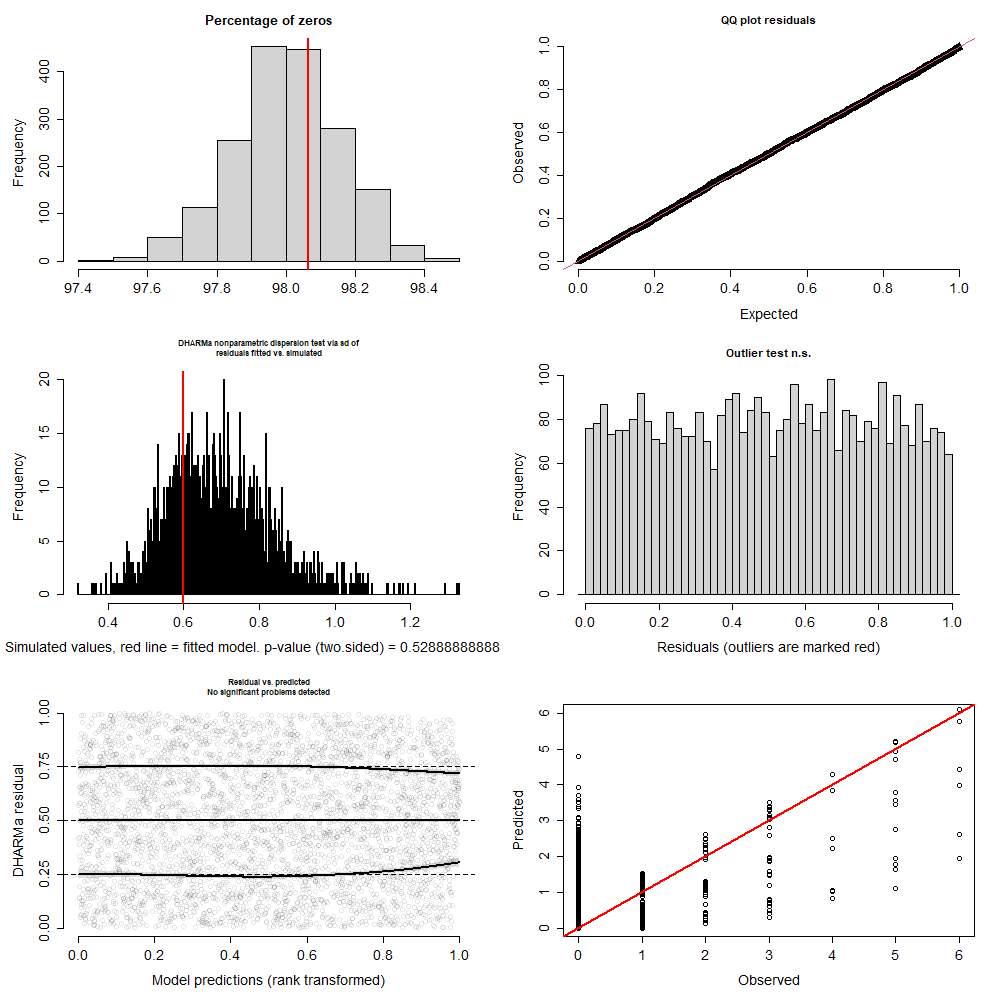
**

**Figure S14.** Dusky dolphin – Model 1. Results for simulation-based and scaled residuals diagnostics for model-fit assessment. Left top panel shows the percentage of zeros in observed data (red line) and simulated data (histogram of posterior distribution). Right top panel show expected versus observed residual plot using scaled residuals. Left mid-panel show residual dispersion for observed data (red line) and simulated data (histogram of posterior distribution). Right mid-panel show result for outlier test, indicating now outliers detected. Left bottom panel show quantile deviation test, red lines indicate deviations detected. Right bottom panel show observed versus predicted plot.

**Table S11.** Results for model 2 fitted to dusky dolphin data. Parameters for predictors are indicated by specific predictors’ name including quadratic terms. Parameters in bold indicate zero was not included in their posterior distribution. Psi (ψ) is the probability of a non-zero and prec corresponds to the precision (1/variance) of the normally distributed observation-level random effect.

| **Dusky dolphin - Model 2** | | | | | | | |
| --- | --- | --- | --- | --- | --- | --- | --- |
|  | **mean** | **sd** | **2.50%** | **25%** | **50%** | **75%** | **97.50%** |
| Intercept | -9.31476 | 1.066228 | -11.3906 | -10.07 | -9.30017 | -8.60226 | -7.26725 |
| **SST** | **1.230694** | **0.243786** | **0.777884** | **1.063106** | **1.220883** | **1.378097** | **1.752263** |
| **SST^2^** | **-0.80824** | **0.207141** | **-1.25515** | **-0.93768** | **-0.79752** | **-0.66582** | **-0.44446** |
| **DEPTH** | **4.116265** | **1.123578** | **2.643973** | **3.372017** | **3.848724** | **4.631435** | **6.946402** |
| DEPTH^2^ | 0.561625 | 0.882892 | -1.65732 | 0.163326 | 0.774268 | 1.166827 | 1.748375 |
| SLO | -0.29342 | 0.253641 | -0.79888 | -0.46766 | -0.28684 | -0.13003 | 0.206738 |
| SLO^2^ | -0.22405 | 0.216342 | -0.68375 | -0.36029 | -0.2041 | -0.06426 | 0.123411 |
| Mean group size | 18.61561 | 1.320562 | 16.19678 | 17.69688 | 18.57137 | 19.44233 | 21.34528 |
| Variance group size | 273.7462 | 48.04307 | 196.2432 | 239.6013 | 268.6572 | 301.7448 | 383.0722 |
| psi | 0.439898 | 0.177076 | 0.198025 | 0.309284 | 0.407106 | 0.528026 | 0.882689 |
| prec | 0.361169 | 0.13785 | 0.227369 | 0.262363 | 0.31788 | 0.414728 | 0.723256 |
| A0 | -0.30579 | 0.086681 | -0.47159 | -0.36154 | -0.30948 | -0.25155 | -0.12254 |
| Wave height | -0.15791 | 0.109307 | -0.37452 | -0.23249 | -0.15839 | -0.08613 | 0.063491 |
| Wind speed | 0.121607 | 0.119395 | -0.12337 | 0.042282 | 0.124986 | 0.20215 | 0.351834 |
| Sea State | -0.1692 | 0.111255 | -0.39335 | -0.24025 | -0.16528 | -0.09532 | 0.040012 |
| bpv | 0.558889 | 0.496658 | 0 | 0 | 1 | 1 | 1 |
| c.hat | 1.942276 | 5.010608 | 0.275975 | 0.846957 | 1.03236 | 1.401231 | 9.335658 |
| observed range | 6 | 0 | 6 | 6 | 6 | 6 | 6 |
| expected range | 7.8 | 1.594529 | 5 | 7 | 8 | 9 | 11 |

**
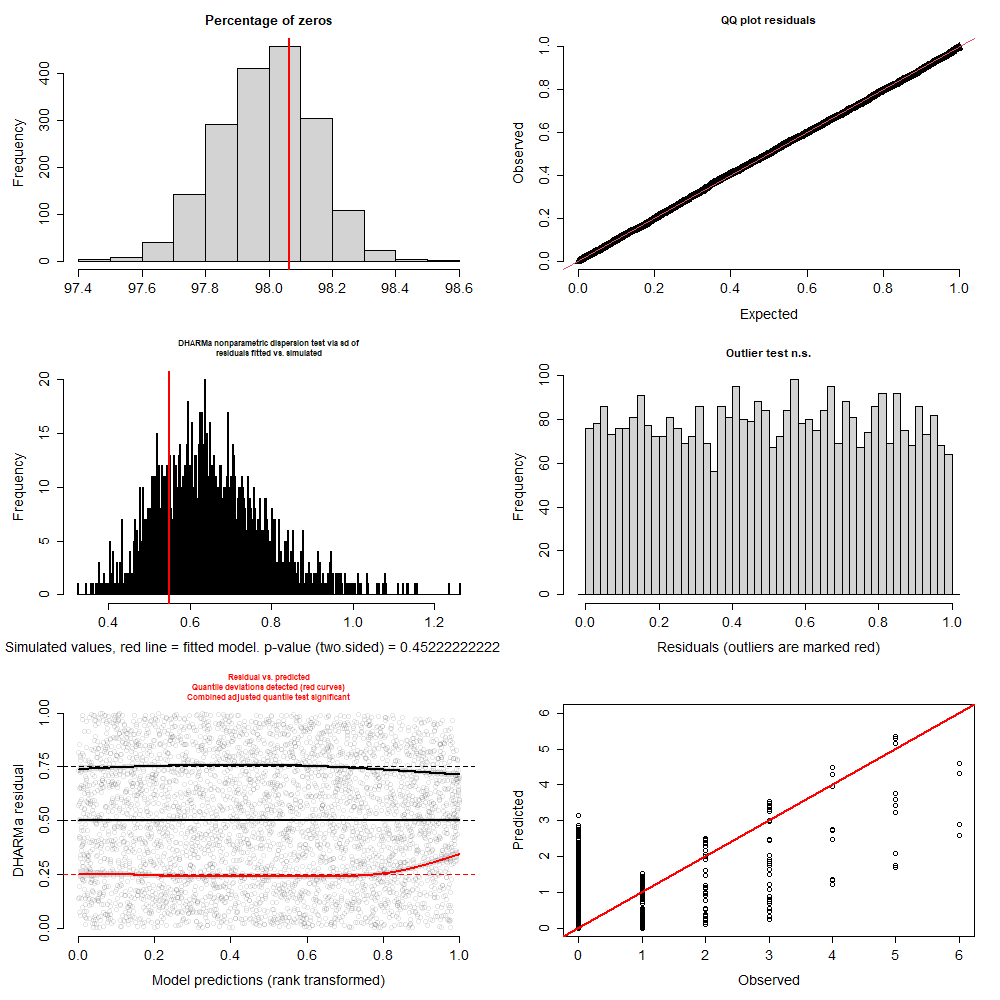
**

**Figure S15.** Dusky dolphin – Model 2. Results for simulation-based and scaled residuals diagnostics for model-fit assessment. Left top panel shows the percentage of zeros in observed data (red line) and simulated data (histogram of posterior distribution). Right top panel show expected versus observed residual plot using scaled residuals. Left mid-panel show residual dispersion for observed data (red line) and simulated data (histogram of posterior distribution). Right mid-panel show result for outlier test, indicating now outliers detected. Left bottom panel show quantile deviation test, red lines indicate deviations detected. Right bottom panel show observed versus predicted plot.

**Table S12.** Results for model 1 fitted to common dolphin data. Parameters for predictors are indicated by specific predictors’ name including quadratic terms. Parameters in bold indicate zero was not included in their posterior distribution. Psi (ψ) is the probability of a non-zero and prec corresponds to the precision (1/variance) of the normally distributed observation-level random effect.

| **Common dolphin - Model 1** | | | | | | | |
| --- | --- | --- | --- | --- | --- | --- | --- |
|  | **mean** | **sd** | **2.50%** | **25%** | **50%** | **75%** | **97.50%** |
| Intercept | -7.0809 | 1.230293 | -8.82602 | -8.11167 | -7.37916 | -6.05202 | -4.71486 |
| **SST** | **0.598169** | **0.227547** | **0.188474** | **0.438282** | **0.589952** | **0.754257** | **1.083275** |
| **SST^2^** | **-0.47711** | **0.185262** | **-0.8462** | **-0.60394** | **-0.47224** | **-0.34964** | **-0.12984** |
| **DEPTH** | **-0.44025** | **0.224233** | **-0.87468** | **-0.58459** | **-0.44166** | **-0.28804** | **-0.01163** |
| DEPTH^2^ | -0.03205 | 0.159317 | -0.33571 | -0.13985 | -0.02822 | 0.076795 | 0.282172 |
| Mean group size | 46.9311 | 5.572096 | 37.75452 | 43.03957 | 46.43673 | 50.13282 | 58.62344 |
| Variance group size | 2214.256 | 667.288 | 1274.206 | 1750.944 | 2094.541 | 2521.984 | 3776.481 |
| psi | 0.52661 | 0.292094 | 0.098062 | 0.244158 | 0.516248 | 0.801111 | 0.984148 |
| prec | 0.954506 | 0.991448 | 0.252117 | 0.35284 | 0.486102 | 1.07334 | 3.912544 |
| A0 | 0.161956 | 0.129493 | -0.08367 | 0.070453 | 0.156713 | 0.24929 | 0.418774 |
| Wave height | -0.1787 | 0.186618 | -0.53747 | -0.3036 | -0.18262 | -0.05231 | 0.193517 |
| Wind speed | -0.30249 | 0.166989 | -0.62746 | -0.4117 | -0.30428 | -0.18719 | 0.020782 |
| Sea State | 0.02188 | 0.168566 | -0.29961 | -0.08985 | 0.021143 | 0.129939 | 0.361796 |
| bpv | 0.444444 | 0.497042 | 0 | 0 | 0 | 1 | 1 |
| c.hat | 1.034627 | 0.56984 | 0.412195 | 0.793051 | 0.964204 | 1.153452 | 2.053162 |
| observed range | 3 | 0 | 3 | 3 | 3 | 3 | 3 |
| expected range | 2.971667 | 0.868687 | 2 | 2 | 3 | 3 | 5 |

**
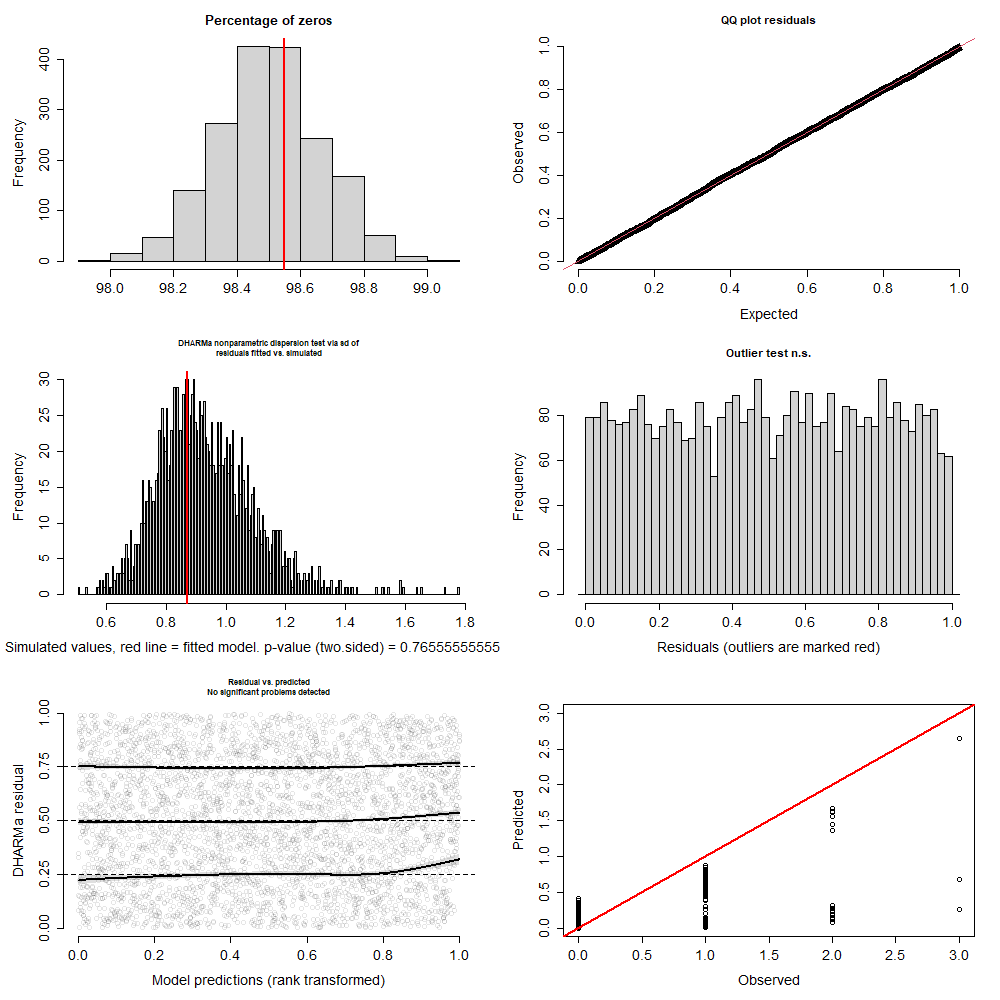
**

**Figure S16.** Common dolphin – Model 1. Results for simulation-based and scaled residuals diagnostics for model-fit assessment. Left top panel shows the percentage of zeros in observed data (red line) and simulated data (histogram of posterior distribution). Right top panel show expected versus observed residual plot using scaled residuals. Left mid-panel show residual dispersion for observed data (red line) and simulated data (histogram of posterior distribution). Right mid-panel show result for outlier test, indicating now outliers detected. Left bottom panel show quantile deviation test, red lines indicate deviations detected. Right bottom panel show observed versus predicted plot.

**Table S13.** Results for model 2 fitted to common dolphin data. Parameters for predictors are indicated by specific predictors’ name including quadratic terms. Parameters in bold indicate zero was not included in their posterior distribution. Psi (ψ) is the probability of a non-zero and prec corresponds to the precision (1/variance) of the normally distributed observation-level random effect.

| **Common dolphin - Model 2** | | | | | | | |
| --- | --- | --- | --- | --- | --- | --- | --- |
|  | **mean** | **sd** | **2.50%** | **25%** | **50%** | **75%** | **97.50%** |
| Intercept | -6.71952 | 0.995821 | -8.85074 | -7.4082 | -6.5222 | -5.93751 | -5.20542 |
| **SSTLT** | **1.192024** | **0.310012** | **0.630611** | **0.981591** | **1.177607** | **1.389304** | **1.854776** |
| SSTLT2 | -0.29639 | 0.239809 | -0.79095 | -0.44648 | -0.279 | -0.13 | 0.138359 |
| DEPTH | -0.28576 | 0.218861 | -0.72928 | -0.42451 | -0.28248 | -0.1408 | 0.144592 |
| DEPTH2 | -0.06954 | 0.153364 | -0.38154 | -0.17097 | -0.0693 | 0.036651 | 0.234916 |
| Mean group size | 46.76879 | 5.548721 | 36.89728 | 42.95904 | 46.24758 | 50.15738 | 58.87203 |
| Variance group size | 2204.686 | 662.6679 | 1264.458 | 1762.38 | 2090.233 | 2525.698 | 3755.97 |
| psi | 0.364786 | 0.2248 | 0.107072 | 0.187872 | 0.290881 | 0.485299 | 0.906012 |
| prec | 1.681376 | 1.087629 | 0.313682 | 0.728465 | 1.443721 | 2.426931 | 4.136162 |
| A0 | 0.149473 | 0.124001 | -0.0749 | 0.060587 | 0.142866 | 0.230754 | 0.411189 |
| Wave height | -0.24785 | 0.178141 | -0.5884 | -0.37156 | -0.25307 | -0.13287 | 0.105355 |
| Wind speed | -0.28459 | 0.162494 | -0.60636 | -0.39624 | -0.28055 | -0.17879 | 0.030288 |
| Sea State | 0.065135 | 0.169394 | -0.2588 | -0.04568 | 0.065631 | 0.172373 | 0.403824 |
| bpv | 0.441111 | 0.496658 | 0 | 0 | 0 | 1 | 1 |
| c.hat | 1.010597 | 0.414946 | 0.469798 | 0.823802 | 0.968477 | 1.135011 | 1.688826 |
| observed range | 3 | 0 | 3 | 3 | 3 | 3 | 3 |
| expected range | 2.829444 | 0.771447 | 2 | 2 | 3 | 3 | 5 |

**
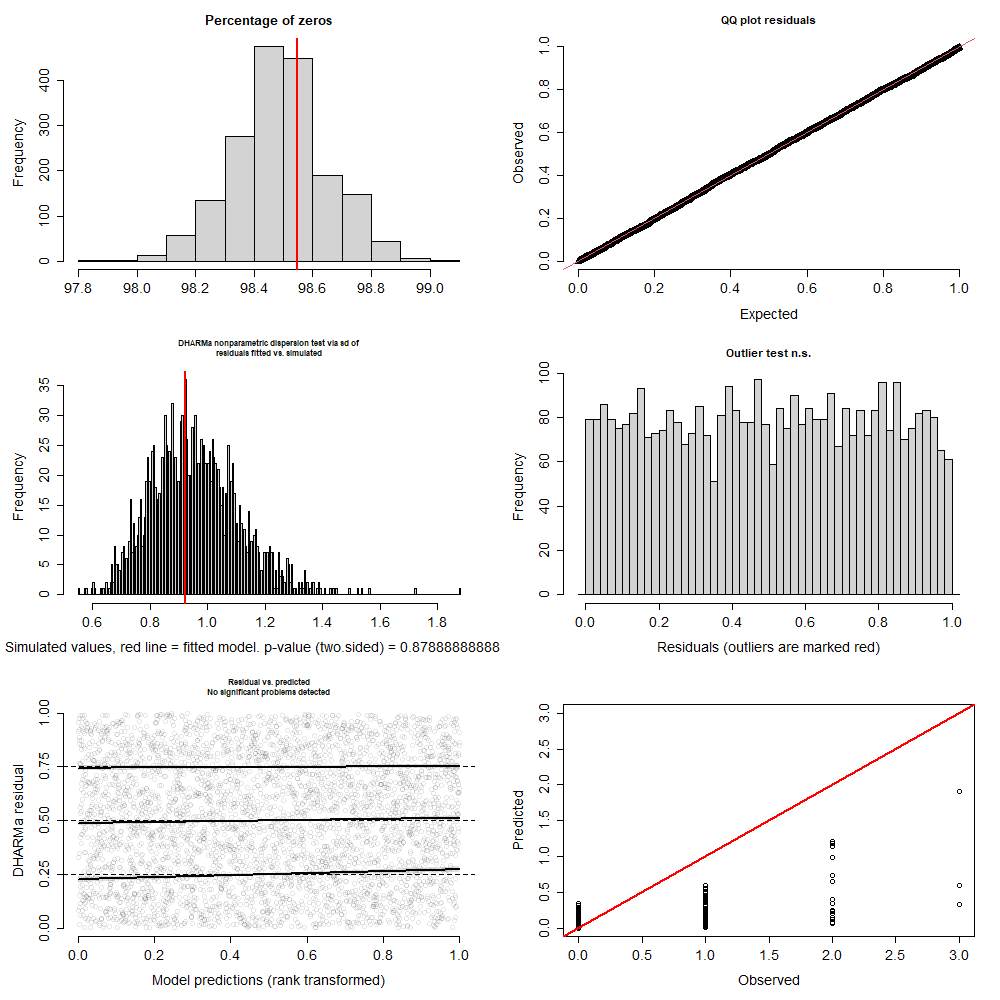
**

**Figure S17.** Common dolphin – Model 2. Results for simulation-based and scaled residuals diagnostics for model-fit assessment. Left top panel shows the percentage of zeros in observed data (red line) and simulated data (histogram of posterior distribution). Right top panel show expected versus observed residual plot using scaled residuals. Left mid-panel show residual dispersion for observed data (red line) and simulated data (histogram of posterior distribution). Right mid-panel show result for outlier test, indicating now outliers detected. Left bottom panel show quantile deviation test, red lines indicate deviations detected. Right bottom panel show observed versus predicted plot.

**
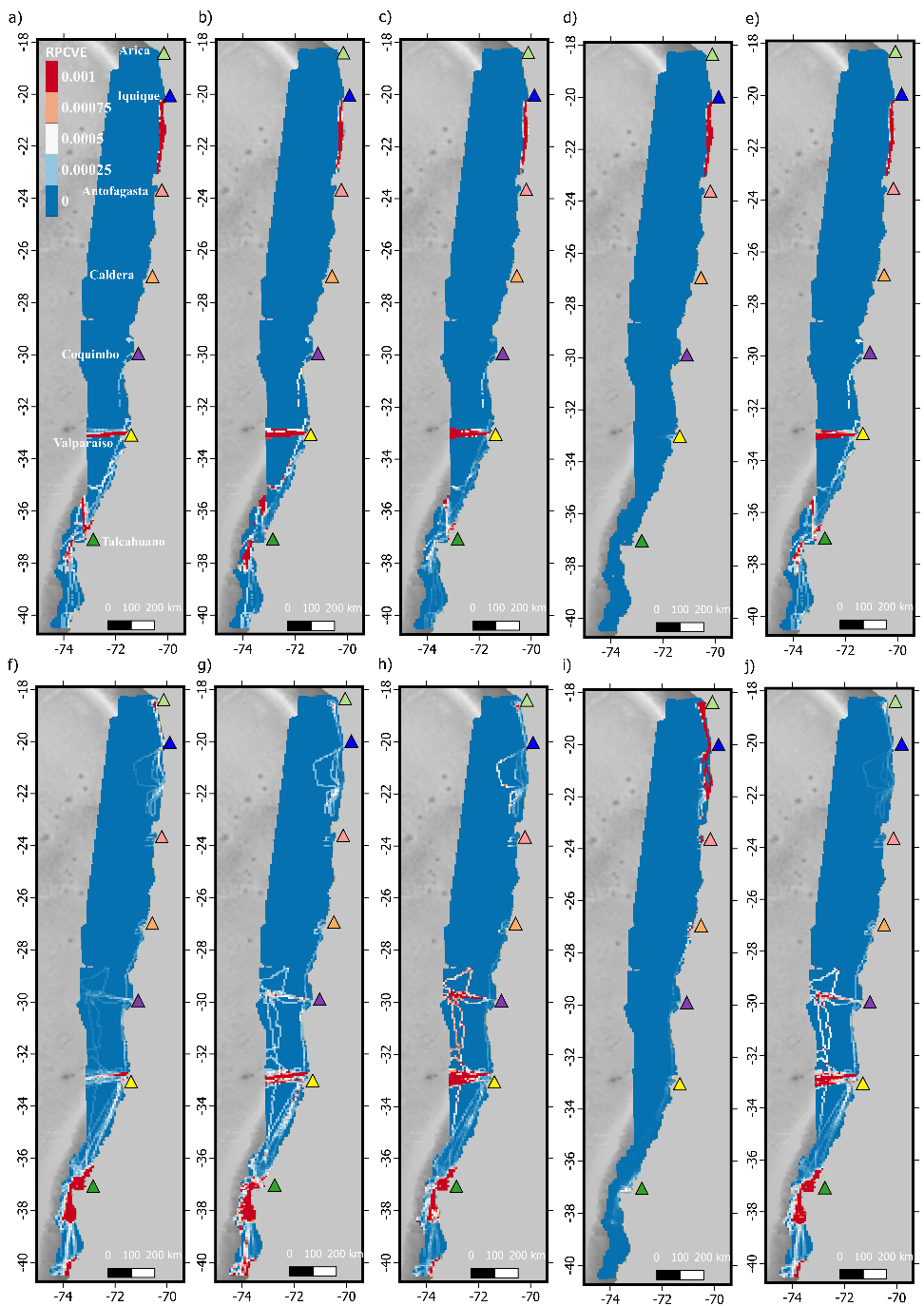
**

**Figure S18.** Predicted relative probability of cetacean-vessel encounter (RPCVE) for transport fleet (a-e) and aquaculture fishery fleet (f-j). Columns show results for fin whales (a,f), blue whales (b,g), sperm whales (c,h), dusky dolphins (d,i) and common dolphins (e,j). Colored triangles indicate the location of most important ports. Data layers (including maps) were created in R ver. 4.0.2 ([www.r-project.org](http://www.r-project.org)) and ensembled in QGIS ver. 3.8.0 ([www.qgis.org](http://www.qgis.org)) for final rendering. Maps were created using data on bedrock topography from the National Centers for Environmental Information (<https://maps.ngdc.noaa.gov/viewers/grid-extract/index.html>). Grid-cells with values above 0 were considered land coverage and assigned a uniform color.

**
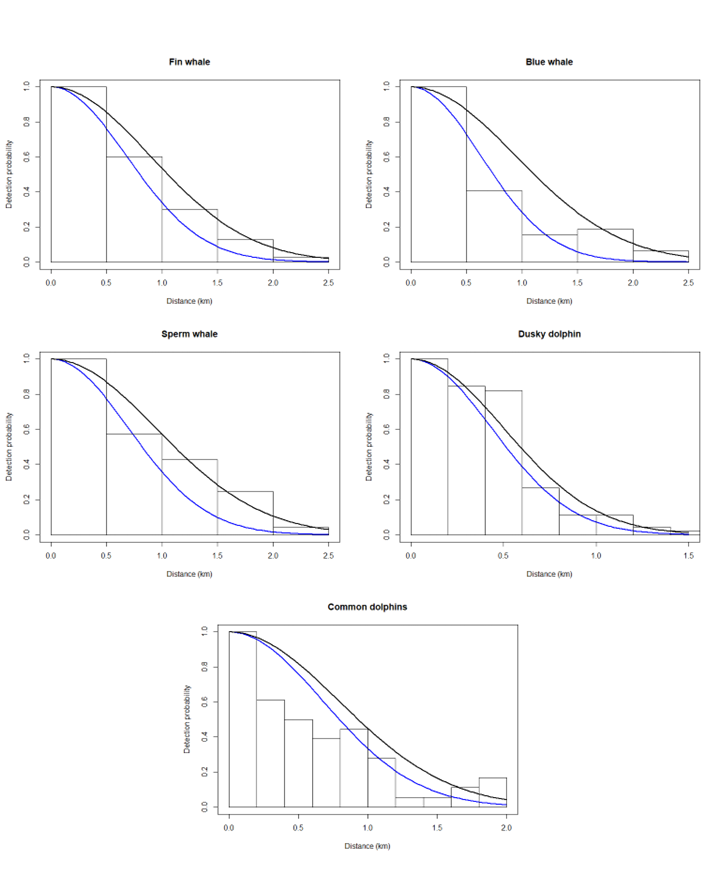
**

**Figure S19.** Detection curves for the five cetacean species in this study. Lines show effect of wave height condition on the detection curve with the black line indicating condition 1 (equal or less than 1.75-2.25 m) and the blue line condition 2 (above 2.25 410 m).

**BUGS code for fitting the Bayesian Binomial N-mixture model**

model{

Priors

#Environmental covariate parameters

for (i in 1:nCovs){

B[i]~ dnorm(0,0.01)

}

#Observation covariate parameters

A0 ~ dnorm(0,0.1)

A1 ~ dnorm(0,0.1)

A2 ~ dnorm(0,0.1)

A3 ~ dnorm(0,0.1)

#Group size priors

r ~ dgamma(0.01,0.01)#size

pg <- r/(r+mug)#prob of success

mug~dgamma(0.01,0.01)#mean

vg<-r*(1-pg)/(pg*pg)#variance

psi ~ dunif(0, 1)

logprec ~ dunif(-1.5,1.5)

prec <-exp(logprec)

#Detections

for (k in 1:nDet){

#Chelgren et al. Likelihood function

Lik[k] <- 1/sigma[k]*pow(2*pi,-0.5)*exp(-0.5*pow(x[k]/sigma[k],2)/(phi(W/sigma[k])-0.5))

sigma[k]<-exp(A0 + A1*wh_d[k]+ A2*ss_d[k]+ A3*ws_d[k])

p[k] <- Lik[k] / C

#The ones trick

ones[k] ~ dbern(p[k])

#Estimating group size

gsize[k]~ dnegbin(pg,r)

}

eta <- Xb %*% B

for(i in 1:UM){

mu[i] <- lambda[i]*z[i]+0.00001

OLRE[i] ~ dnorm(0, prec)

lambda[i] <- 2*W*L[i]*exp(eta[i]+ OLRE[i]) #OLRE

z[i] ~ dbern(psi)

N[i] ~ dpois(mu[i])

sigma_t[i]<-exp(A0 + A1*wh[i]+ A2*ss[i]+ A3*ws[i])

tau[i] <- pow(sigma_t[i],-2)

Pn[i]<- (1/W)*sqrt(2*pi/tau[i])*(phi(W*sqrt(tau[i]))-0.5)

n[i] ~ dbin(Pn[i],N[i])

e.count[i] <- lambda[i]*Pn[i]*psi # Expected datum

n.new[i]~ dbin(Pn[i],N[i])# Create new data set under model

resi[i]<-(n[i]-e.count[i]) / (sqrt(e.count[i])+0.00001) # Pearson residual

chi2[i] <- pow((n[i]-e.count[i]),2) / (e.count[i]+0.00001) # obs.

chi2.new[i] <- pow((n.new[i]-e.count[i]),2) / (e.count[i]+0.00001) # exp.

}

#Add up discrepancy measures for entire data set

fit <- sum(chi2[]) # Omnibus test statistic actual data

fit.new <- sum(chi2.new[]) # Omnibus test statistic replicate data

c.hat <- fit / fit.new # c-hat estimate

bpv <- step(fit-fit.new) # Bayesian p-value

#range of data as a second discrepancy measure

obs.range <- max(n[]) - min(n[])

exp.range <- max(n.new[]) - min(n.new[])

#Constants

C <- 100000

pi <- 3.14159

}
